# Ferulic acid-loaded chitosan nanoparticles enhance radiotherapy efficacy via STAT3 suppression and caspase-8/p53 activation in Ehrlich ascites carcinoma

**DOI:** 10.1038/s41598-025-27352-8

**Published:** 2025-12-01

**Authors:** Mai Tamer Mansour, Mohammed Abdalla Hussein, Ahmed A. Emara, Rabab M. El-Sherif

**Affiliations:** 1 Technology of Radiology and Medical Imaging Department, Faculty of Applied Health Science Technology, October 6 University, October 6th City, Egypt; 2 Department of Biotechnology, Faculty of Applied Health Sciences Technology, October 6 University, October 6th City, Egypt; 3 Faculty of Postgraduate Studies for Nanotechnology, Cairo University, Cairo, Egypt

**Keywords:** Chitosan nanoparticles, Ferulic acid, Radiosensitization, STAT3, Caspase-8, p53, Biochemistry, Cancer, Drug discovery

## Abstract

Radiotherapy resistance remains a major challenge in cancer treatment, necessitating novel strategies to enhance therapeutic efficacy while minimizing side effects. This study aimed to develop and evaluate ferulic acid-loaded chitosan nanoparticles (FA-ChNPs) as a multimodal radiosensitizer targeting STAT3, caspase-8, and p53 pathways in Ehrlich ascites carcinoma (EAC). FA-ChNPs were synthesized using ionic gelation and thoroughly characterized for their physicochemical properties, including size, and zeta potential through TEM and DLS. The encapsulation efficiency was verified using UV–Vis spectroscopy. In vitro drug release kinetics were evaluated using the dialysis bag method, demonstrating a sustained release profile from the nanoparticles. Biological evaluation included acute toxicity testing in healthy mice to determine the LD50 value according to OECD guidelines. For antitumor assessment, EAC-bearing mice were divided into six treatment groups (n equals 6 per group), receiving either FA-ChNPs alone (115.75mg/kg via oral gavage), gamma-irradiation alone (6Gy per week), or combination therapy. Comprehensive biological parameters were measured, including tumor progression (mass and volume), metabolic profile (total cholesterol, triglycerides, HDL-C), liver and kidney function markers (ALT, AST, ALP, creatinine, urea), oxidative stress indicators (GSH, SOD, CAT, MDA), inflammatory cytokines (TNF-alpha, IL-6, VEGF), and gene expression levels (STAT3, caspase 8, P53 via qRT-PCR). Histopathological examination of liver tissue complemented these analyses. In silico studies encompassed molecular docking with key targets (STAT3, caspase 8, TP53) and ADMET property predictions. The FA-ChNPs demonstrated favorable physicochemical properties, including a spherical morphology with an average particle size of 41.63 nm and good colloidal stability indicated by a PDI of 0.2, a zeta potential of -2.45 mV. Also, FA-ChNPs exhibited sustained in vitro drug release, contrasting with the rapid diffusion of free ferulic acid, which supports their potential for prolonged therapeutic action and a high safety margin with an LD₅₀ value of 2315mg/kg. The combination therapy demonstrated superior antitumor effects, achieving 44.3% reduction in tumor mass and 67.0% reduction in tumor volume compared to EAC controls, with corresponding tumor dimensions reduced to 1.02cm (length) × 0.74cm (width), confirming potent radiosensitization and synergistic efficacy. Biological assessments revealed significant improvements across multiple parameters: metabolic profile showed 89.2% increase in HDL-C and 29.0% decrease in triglycerides; liver and kidney function markers improved with 29.0% reduction in ALT and 39.7% decrease in creatinine; oxidative stress modulation included 87.9% increase in GSH and 32.4% reduction in MDA; inflammatory markers decreased by 40% for TNF-alpha and 51.1% for IL-6. Molecular analyses showed 70% suppression of STAT3, 108.2% activation of caspase-8, and stabilization of p53. Histopathological evaluation confirmed preserved liver architecture with minimal steatosis and inflammation. Molecular docking studies revealed strong binding affinities:—6.02 kcal/mol for STAT3, -7.31kcal/mol for CASP8, and -5.15kcal/mol for TP53. FA-ChNPs represent a groundbreaking approach to enhance radiotherapy efficacy through simultaneous pathway modulation and organoprotection. These findings support further clinical translation for treatment-resistant cancers.

**Supplementary Information:**

The online version contains supplementary material available at 10.1038/s41598-025-27352-8.

## Introduction

Cancer represents one of the most significant challenges to global health, characterized by uncontrolled cellular proliferation and metastatic potential. As the second leading cause of death worldwide^1^, cancer accounted for approximately 10 million fatalities in 2020, with projections suggesting a 47% increase in incidence by 2040^2^. In Egypt, the National Cancer Registry Program reports about 134,000 new cases annually, with hepatocellular carcinoma (23.8%), breast cancer (15.2%), and bladder cancer (6.9%) being most prevalent^3^. These alarming statistics underscore the urgent need for more effective therapeutic strategies^4^.

Radiotherapy remains a cornerstone of cancer treatment, employed in over 50% of cases either as primary or adjuvant therapy^5^. However, its efficacy is frequently limited by tumor radioresistance—a multifactorial phenomenon involving hypoxia, enhanced DNA repair, and activation of pro-survival pathways like STAT3^6^. This resistance necessitates higher radiation doses that often damage healthy tissues, creating a critical need for effective radiosensitizers^7^.

Natural products have emerged as promising radiosensitizing agents due to their multi-target effects and favorable safety profiles^8^. Ferulic acid, a ubiquitous plant phenolic compound, exhibits remarkable anticancer properties through multiple mechanisms^9^. Despite these advantages, ferulic acid’s clinical application is hampered by poor pharmacokinetics, including rapid metabolism and limited tumor accumulation^10^. Nanotechnology offers an innovative solution to these challenges^11^. Chitosan-based nanoparticles, in particular, provide numerous advantages for drug delivery; enhanced permeability and retention (EPR) effect, pH-responsive drug release in tumor microenvironments, improved cellular uptake and bioavailability and reduced systemic toxicit ^12^.

Chitosan nanoparticles enhance tumor radiosensitivity by facilitating targeted delivery of therapeutic agents^13^. They increase intracellular drug accumulation and amplify radiation-induced DNA damage. Their mucoadhesive and cationic nature improves tumor penetration and cellular uptake^14^. Functionalized ChNPs modulate oxidative stress and apoptosis pathways in cancer cells. This synergistic effect allows for lower radiation doses with improved therapeutic outcomes^15^.

Overexpression of STAT3 enhances tumor resistance to radiotherapy by promoting DNA repair, inhibiting apoptosis, and sustaining cancer stem cell survival^16^. In contrast, mutant or dysfunctional p53 impairs radiation-induced cell cycle arrest and apoptosis, allowing damaged cells to persist^17^. Loss of p53 function also disrupts transcriptional control of pro-apoptotic genes^18^. Meanwhile, downregulation of caspase-8 compromises the extrinsic apoptotic pathway, reducing sensitivity to death receptor-mediated signals triggered by radiation^19^. Together, these alterations create a pro-survival environment that shields tumor cells from radiotherapy-induced cytotoxicity. Targeting these pathways may restore radiosensitivity and improve therapeutic outcomes^15^.

Ferulic acid-chitosan nanoparticles (FA-ChNPs) act as a multimodal radiosensitizer through three synergistic mechanisms: enhancing radiation-induced DNA damage by suppressing cytokine-mediated survival signaling and reprogramming the tumor immune microenvironment through dual cytokine modulation-downregulating radioresistance-associated cytokines while upregulating radiosensitizing cytokines^20–22^. This tripartite action promotes radiation-induced apoptosis while protecting normal tissues, potentially reducing the required therapeutic radiation dose by 30–40% in Ehrlich carcinoma models^23–27^.

While several nano-formulations have been explored as radiosensitizers, their action is often limited to a single pathway, such as enhancing oxidative stress or drug delivery. The novelty of the FA-ChNP platform presented here lies in its multimodal strategy, designed to concurrently dismantle multiple pillars of radioresistance. This approach simultaneously targets the STAT3-driven pro-survival signaling, reactivates the extrinsic apoptotic pathway via caspase-8, and stabilizes the tumor suppressor p53. Ferulic acid is the key bioactive agent enabling this coordinated attack; it has been shown to inhibit STAT3 phosphorylation and nuclear translocation, to promote the activation of caspase-8, and to facilitate p53 stabilization and its transcriptional activity. By co-delivering ferulic acid in a chitosan nanocarrier that enhances tumor accumulation and provides a controlled release, we hypothesize that FA-ChNPs can achieve a synergistic radiosensitizing effect that is unattainable with single-target agents or free ferulic acid alone.

This investigation aims to evaluate the radiosensitizing potential of FA-ChNPs in Ehrlich ascites carcinoma models and characterize the molecular mechanisms through STAT3, p53 and caspase-8 modulation.

## Materials and methods

Ferulic acid (≥ 98%) was purchased from Sigma-Aldrich (USA), while chitosan (medium molecular weight, ~ 200–300 kDa) was obtained from Shanghai Macklin Biochemical Co., Ltd. (China). Tween-80 was supplied by Jiangsu Fengyuan Biotechnology Co., Ltd. (China). Additional reagents, including washing buffer, blocking buffer, diluent buffer, and substrate buffer (Sigma-Aldrich, Cat. No. T7517-5G), were also used.

### Cell culture and treatment

Ehrlich ascites carcinoma (EAC) cells were obtained from the National Cancer Institute, Cairo University (Egypt). The cells were maintained in RPMI-1640 medium supplemented with 10% fetal bovine serum (FBS) and 1% penicillin/streptomycin at 37°C in a humidified 5% CO₂ atmosphere. Seven days prior to treatment with ferulic acid nanoparticles, mice received intraperitoneal injections of EAC cells (2.5 × 10⁶ cells/mouse).

## Methods

### Synthesis of ferulic acid- chitosan nanoparticles

FA-ChNPs were prepared via ionic gelation using an optimized protocol. Initially, chitosan (low molecular weight, 1g, 85% deacetylation) and calcium chloride (0.01g) were dissolved in 100mL of distilled water (pH 5.2 adjusted with 0.1M acetic acid) under magnetic stirring (1200rpm) at 70°C for 45min to ensure complete polymer dissolution^28^. Glycerol (0.5mL, 50% v/w) was then incorporated as a plasticizer with continued stirring at 70°C for 10min. The solution was cooled to 55°C, followed by sequential addition of ferulic acid (8 μg/mL, dissolved in 1mL ethanol) and Tween 80 (8μg/mL) as a surfactant. The mixture was immediately homogenized (Heidolph Silent Crusher M, Germany) at 13,500 rpm for 3min at 25°C to form a stable nanoemulsion. Blank chitosan nanoparticles (ChNPs) were prepared identically without ferulic acid as a control. For drying, 25 mL aliquots of the nanoparticle suspension were cast onto Teflon plates (10cm diameter) and dried at 37°C (40% relative humidity) for 30h. The resulting films were carefully peeled, vacuum-desiccated (25°C, 0.1 bar), and stored in light-protected containers until further characterization.

### Characterization of ferulic acid nanoparticles

#### Morphological analysis

The surface morphology and shape of the ferulic acid-loaded chitosan nanoparticles (FA-ChNPs) were evaluated using both Transmission Electron Microscopy (TEM) and Scanning Electron Microscopy (SEM). For TEM analysis, a Tecnai 20 G2 S TWIN microscope (IIT Roorkee) operated at an accelerating voltage of 200 kV was used. Air-dried nanoparticle suspensions were deposited onto Formvar-coated copper grids for imaging. For SEM analysis, a Quanta FEG 250 microscope (FEI, Netherlands) was employed. A drop of the diluted nanoparticle suspension was placed on an aluminum stub, allowed to air-dry, and then sputter-coated with a thin layer of gold to enhance conductivity prior to imaging..

#### Particle size and zeta potential analysis

FA-ChNPs were characterized for hydrodynamic diameter, polydispersity index (PDI), and zeta potential using dynamic light scattering (Malvern Zetasizer Nano ZS90). Samples were diluted 20-fold in distilled water and measured in triplicate at 25°C. Zeta potential was determined via electrophoretic light scattering with 1 mM NaCl maintaining 50 μS/cm conductivity^29^. Blank chitosan nanoparticles (ChNPs), synthesized using the same ionic gelation method but without the addition of ferulic acid, were also characterized under identical conditions to serve as a control.

#### FT-IR spectroscopy

Fourier-transform infrared (FT-IR) spectra were acquired using a Vertex 70 infrared spectrophotometer (Bruker, Karlsruhe, Germany) following the method described by Dickinson. Each sample (1mg) was thoroughly mixed with 100mg of KBr, compressed into a pellet, and scanned over the 400–4000cm⁻^1^ range at a resolution of 4cm⁻^1^ with 64 scans per sample.

#### UV–Vis spectroscopy

UV–visible spectra of both Ferulic acid nanoparticles and free Ferulic acid were recorded using a spectrophotometer (Jasco, Japan) in the wavelength range of 200–700nm with 1.0nm intervals. All measurements were conducted at 25°C in 1cm path length quartz cuvettes and averaged over triplicate runs.

#### In vitro drug release study

The in vitro release profile of ferulic acid from the FA-ChNPs was investigated using the dialysis bag method under sink conditions. Briefly, a suspension of FA-ChNPs equivalent to 2mg of ferulic acid was placed in a dialysis bag (Cellulose Membrane, Molecular Weight Cut-Off: 12kDa, Sigma-Aldrich). The bag was securely sealed and immersed in 100mL of phosphate-buffered saline (PBS, 0.1M, pH 7.4) as the release medium, maintained at 37 ± 0.5°C under continuous magnetic stirring at 100rpm. At predetermined time intervals (0, 1, 2, 4, 6, 8, 10, 12, and 25 h), 1 mL aliquots of the release medium were withdrawn and replaced immediately with an equal volume of fresh, pre-warmed PBS to maintain the sink condition. The concentration of ferulic acid in the withdrawn samples was quantified using a pre-calibrated UV–Vis spectrophotometer (Jasco, Japan) at the wavelength of 332nm. The cumulative percentage of drug release was calculated using a standard calibration curve. For comparison, an equivalent amount of free ferulic acid solution was analyzed under identical conditions. All experiments were performed in triplicate, and the results are expressed as mean ± standard deviation (SD).

#### Animals and housing conditions

A total of 96 adult male mice (8–10 weeks old, weighing 35.5 ± 3g) was procured from the National Cancer Institute. The cohort will be divided into two experimental groups: 90 mice for LD₅₀ determination of ferulic acid-chitosan nanoparticles (FA-ChNPs), and 36 mice for antitumor efficacy evaluation in Ehrlich ascites carcinoma (EAC)-bearing models. All animals will be individually housed in sterilized polypropylene cages under controlled environmental conditions: 12:12 h light–dark cycle (08:00–20:00), temperature maintained at 22 ± 1°C, and relative humidity at 55 ± 5%. Following a 7-day acclimatization period with ad libitum access to irradiated rodent chow and UV-treated water, experimental procedures will commence.

#### Determination of LD₅₀

The LD₅₀ of FA-ChNPs will be assessed using the Spearman-Karber method^30^. Mice (n = 10 per dose group) will receive a single oral administration of FA-ChNPs at logarithmically spaced doses (e.g., 100, 200, 400, 800mg/kg). Mortality and signs of toxicity (e.g., lethargy, respiratory distress) will be monitored every 6 h for the first 24 h and daily thereafter for 14 days. The LD₅₀ will be calculated using the formula:

$${\text{LD}}_{{{5}0}} = {\text{Dm }} - \, \left[ { \, \sum \, \left( {{\text{z }} \times {\text{ d}}} \right) \, /{\text{ n}}} \right]$$

**where:**

- ***Dm***: The dose that killed all the mice in the group.
- ***Z***: Half the sum of the dead rats from two successive groups.
- ***d***: The difference between two successive doses.
- ***n***: The number of animals in each group.

#### Animal welfare, humane endpoints, and euthanasia

Animal welfare was monitored twice daily throughout the study. The following humane endpoints were strictly observed, and any animal reaching one or more of these criteria was scheduled for immediate euthanasia to prevent unnecessary suffering:

- Tumor burden exceeding 10% of body weight or a tumor volume > 1500mm^3^.
- Severe lethargy, unresponsiveness to gentle stimuli, or inability to access food and water.
- Difficulty breathing or signs of severe distress.
- Ulceration or necrosis of the tumor that impaired mobility or led to infection.
- Loss of more than 20% of initial body weight.

On the scheduled experimental endpoint (day 36), all remaining mice were euthanized. This was performed via an overdose of sodium thiopental (150mg/kg, intraperitoneal injection), which is more than twice the anesthetic dose, ensuring deep anesthesia followed by cardiac arrest as a confirmatory method. This method is consistent with the AVMA Guidelines for the Euthanasia of Animals. No animals reached the humane endpoint criteria prior to the scheduled time of sacrifice.

#### Experimental design

Ehrlich Ascites Carcinoma (EAC) cells were obtained from the National Cancer Institute, Cairo, Egypt. Tumor maintenance was performed through serial intraperitoneal passages (2.5 × 10⁶ cells/mouse) in male albino mice. For the experimental protocol, 36 mice were randomly allocated into six groups (n = 6 per group) consisting of three control and three therapeutic intervention groups (Table 1). The therapeutic dose of FA-ChNPs (115.75mg/kg) was selected as 1/20th of the determined LD₅₀ (2315mg/kg), a conversion factor recommended by OECD guidelines for sub-acute toxicity and efficacy studies in rodents, ensuring a high safety margin for the prolonged treatment schedule.

**Table 1:** Experimental group design and treatment protocols.

Group	Designation	Treatment Protocol	Purpose
I	Naïve Control	• 3 mL distilled water, oral gavage daily for 5 weeks	Establish baseline physiological parameters
II	FA-ChNP Control	• FA-ChNPs (115.75mg/kg) suspended in distilled water, oral gavage daily for 4 weeks	Evaluate nanoparticle safety and non-tumor effects
III	EAC Tumor Control	• Subcutaneous EAC inoculation (2.5 × 10⁶ cells/mouse), one week before the beginning of experimental	Monitor natural tumor progression
IV	Radiotherapy Control	• EAC inoculation + γ-irradiation (6Gy/week starting day 14) for 3 weeks ^31^ (Egyptian Atomic Energy Authority, Cairo, Egypt)	Assess radiation-only effects
V	FA-ChNPs Therapy	• EAC inoculation + FA-ChNPs (115.75mg/kg) starting day 7 for 4 weeks	Evaluate nanoparticle monotherapy efficacy
VI	Combination Therapy	• EAC inoculation + FA-ChNPs (as Group V) + γ-irradiation (as Group IV) for 4 weeks	Investigate potential synergistic effects of combined treatment

On the 36^th^ day, following 24 h of treatment, all mice were anesthetized by intraperitoneal (i.p.) injection of sodium thiopental (75mg/kg) and sacrificed.

Following euthanasia, solid Ehrlich ascites carcinoma (EAC) tumors were surgically excised, rinsed with ice-cold PBS (pH 7.4), and blotted on sterile filter paper to remove surface moisture. The orthogonal diameters (length and width) of each tumor were measured using digital calipers (Mitutoyo 500–196-30, ± 0.01 mm accuracy) and are reported in centimetres (cm). Tumor mass was measured using an analytical balance (Sartorius CPA225D, ± 0.1mg precision)^32^. Tumor volume was calculated from orthogonal diameters (length [L], width [W]) obtained with digital calipers (Mitutoyo 500–196-30, ± 0.01 mm accuracy) using the modified ellipsoid formula:

$${\text{V }} = \, \left( {{\text{L }} \times {\text{ W}}} \right)/{2}$$

as established for murine tumor models. Necrotic regions (> 2 mm diameter) were excluded from measurements. All procedures complied with NCCLS guidelines for preclinical tumor assessment^33^.

#### Biochemical analysis methodology

Following euthanasia, blood was promptly obtained via cardiac puncture using heparin-coated syringes and transferred to chilled tubes containing EDTA as an anticoagulant. The specimens were then centrifuged at 3000 × g for 15 min at 4°C (Eppendorf 5810R) to isolate plasma. Plasma aliquots were subsequently stored at − 80°C pending biochemical analysis.

#### Lipid profile analysis

Plasma total cholesterol (TC), triglycerides (TG), and high-density lipoprotein cholesterol (HDL-c) were quantified enzymatically using standardized commercial kits (Roche Diagnostics) following established protocols. TC was measured via cholesterol oxidase-p-aminophenazone (CHOD-PAP) method, TG by glycerol-3-phosphate oxidase (GPO-PAP) assay, and HDL-c through phosphotungstic acid precipitation, as validated by the NIH Lipid Research Clinics Program^34^. All assays were performed in triplicate on a Cobas c501 analyzer with NIST-traceable calibrators, maintaining inter-assay coefficients of variation < 3%^35^. Methodology adhered to the International Federation of Clinical Chemistry guidelines for lipid measurement reliability^36^.

#### Hepatic and renal function assessment

Liver function was evaluated by measuring alanine aminotransferase (ALT) and aspartate aminotransferase (AST) activities using International Federation of Clinical Chemistry (IFCC)-approved methods without and with pyridoxal phosphate cofactor, respectively^37^. Alkaline phosphatase (ALP) activity was determined via p-nitrophenylphosphate hydrolysis at 37°C^38^. Renal function was assessed through: (1) creatinine quantification by the modified Jaffé kinetic method (alkaline picrate)^39^, and (2) urea nitrogen measurement using the urease-Berthelot reaction^40^. All assays were performed on a Cobas c501 analyzer (Roche Diagnostics) with NIST-traceable calibrators and quality controls, maintaining inter-assay CV < 3%.

#### Liver oxidative stress analysis

Following euthanasia, livers from Ehrlich ascites carcinoma (EAC)-bearing mice were immediately excised, rinsed in ice-cold saline (0.9% NaCl), blotted dry, and homogenized (10% w/v) in phosphate-buffered saline (PBS, pH 7.4) using a glass-Teflon homogenizer at 4°C. The homogenate was centrifuged (10,000 × g, 15 min, 4°C) and divided for analysis of oxidative stress markers and cytokines. Oxidative stress was assessed by measuring reduced glutathione (GSH) via Ellman’s method^41^, superoxide dismutase (SOD) activity by pyrogallol autoxidation inhibition^42^, catalase (CAT) activity through H₂O₂ decomposition^43^, and malondialdehyde (MDA) using thiobarbituric acid reaction^44^. All assays were performed in sextuplicate with appropriate controls.

#### Cytokine quantification

Pro-inflammatory cytokines were quantified using commercial ELISA kits from three manufacturers: TNF-α (Cat. No. E-EL-M0049, Elabscience Biotechnology, China; Cat. No. ab208348, Abcam, UK; Cat. No. 500849, Cayman Chemical, USA), IL-6 (Cat. No. E-EL-M0044, Elabscience; Cat. No. ab222503, Abcam; Cat. No. 500811, Cayman), and VEGF (Cat. No. E-EL-M0323, Elabscience; Cat. No. ab100751, Abcam; Cat. No. 500840, Cayman). All assays were performed according to manufacturers’ protocols in six replicates, with absorbance measured on a microplate reader. Internal controls validated assay performance, and results were normalized to tissue weight.

#### Quantitative real-time PCR analysis of hepatic STAT3, CASP8, and TP53 expression

The second part of liver was designated for quantifying mRNA expression of signal transducer and activator of transcription 3 (STAT3), caspase-8 (CASP8), and tumor protein p53 (TP53) using quantitative real-time polymerase chain reaction (qRT-PCR). Total RNA was isolated using the RNeasy Mini Kit (Qiagen, Cat. No. 74104), and 1 µg of RNA was reverse transcribed into cDNA using the iScript™ cDNA Synthesis Kit (Bio-Rad, Cat. No. 1708891) according to the manufacturer’s instructions.

qRT-PCR was conducted with SYBR® Green Master Mix (Bio-Rad, Cat. No. 1725120) on a CFX96 Touch™ Real-Time PCR Detection System (Bio-Rad). The amplification protocol included an initial denaturation at 95°C for 10 min, followed by 40 cycles of 95°C for 15 s and 60°C for 60 s. Specific primer pairs used for gene amplification were as follows (Table 2):

**Table 2:** Primer Sequences and amplicon characteristics for target and reference genes used in qRT-PCR.

Gene	Forward Primer (5′ → 3′)	Reverse Primer (5′ → 3′)	Accession No	Amplicon Size
STAT3	CTTTGAGACCGAGGTGTATCACC	GGTCAGCATGTTGTACCACAGG	NM_139276	120 bp
CASP8	AGAAGAGGGTCATCCTGGGAGA	TCAGGACTTCCTTCAAGGCTGC	NM_001080125	110 bp
TP53	CCTCAGCATCTTATCCGAGTGG	TGGATGGTGGTACAGTCAGAGC	NM_011640	130 bp
GAPDH	AGGTCGGTGTGAACGGATTTG	TGTAGACCATGTAGTTGAGGTCA	NM_008084	120 bp

Relative expression levels were determined using the 2^−ΔΔCt^ method, with *GAPDH* serving as the reference gene. Melt curve analysis confirmed the specificity of each amplicon. All reactions were performed in triplicate (n = 3) to ensure accuracy and reproducibility of gene expression data.

#### Histological assessment of liver tissue

Liver tissue samples were fixed in 10% neutral-buffered formalin (pH 7.4) for 24-48h, processed through graded ethanol and xylene, and embedded in paraffin. Sections (4–5 μm) were stained with hematoxylin and eosin (H&E) following standard protocols^45^ and evaluated by a blinded pathologist using an Olympus BX53 microscope (100–400 ×). Histological scoring assessed steatosis (0–3), inflammation (Ishak system), and necrosis percentage, with digital images captured using cellSens software (v1.18) for semiquantitative analysis.

#### In Silico studies of ferulic acid

##### Molecular docking studies

The binding interactions of ferulic acid (PubChem CID: 445,858) with STAT3 (PDB: 1bg1), CASP8 (PDB: 3h11), and TP53 (PDB: 4mzi) were investigated using AutoDock Vina (v1.1.2). Ligand preparation included energy minimization (MMFF94 force field), protonation at pH 7.4, and PDBQT conversion (AutoDock Tools v1.5.6). Protein structures were prepared by removing heteroatoms, adding polar hydrogens, and assigning Kollman charges. Docking grids (50 × 50 × 50 Å^3^) were centered on active sites: STAT3 (Tyr705 phosphorylation pocket), CASP8 (Cys285 catalytic domain), and TP53 (DNA-binding region). The top poses were selected based on binding energy (kcal/mol) and interaction profiles (hydrogen bonds, hydrophobic contacts) analyzed in Discovery Studio Visualizer^46,47^.

##### ADMET prediction

Pharmacokinetic and toxicity profiles of ferulic acid were predicted using the ADMETlab 2.0 and SwissADME platforms. Parameters such as absorption (HIA, Caco-2 permeability), distribution (BBB penetration, plasma protein binding), metabolism (CYP450 inhibition), excretion, and toxicity (AMES test, LD₅₀) were evaluated to assess drug-likeness and safety^48^.

##### Statistical analysis

All data were presented as mean ± standard deviation (SD). Biochemical assays were analyzed with a sample size of *n* = 6 per group, while gene expression studies used *n* = 3 biological replicates. Statistical comparisons were performed using one-way ANOVA followed by Tukey’s post hoc test. Data normality and variance homogeneity were assessed using the Shapiro–Wilk and Levene’s tests, respectively. Significance was accepted at *P* < 0.05. All analyses were conducted using IBM SPSS Statistics, version 21 (IBM Corp., Armonk, NY, USA), with distinct superscript letters denoting statistically different groups.

## Results

### Characterization of synthesized FA-ChNPs

**Fig. 1:**
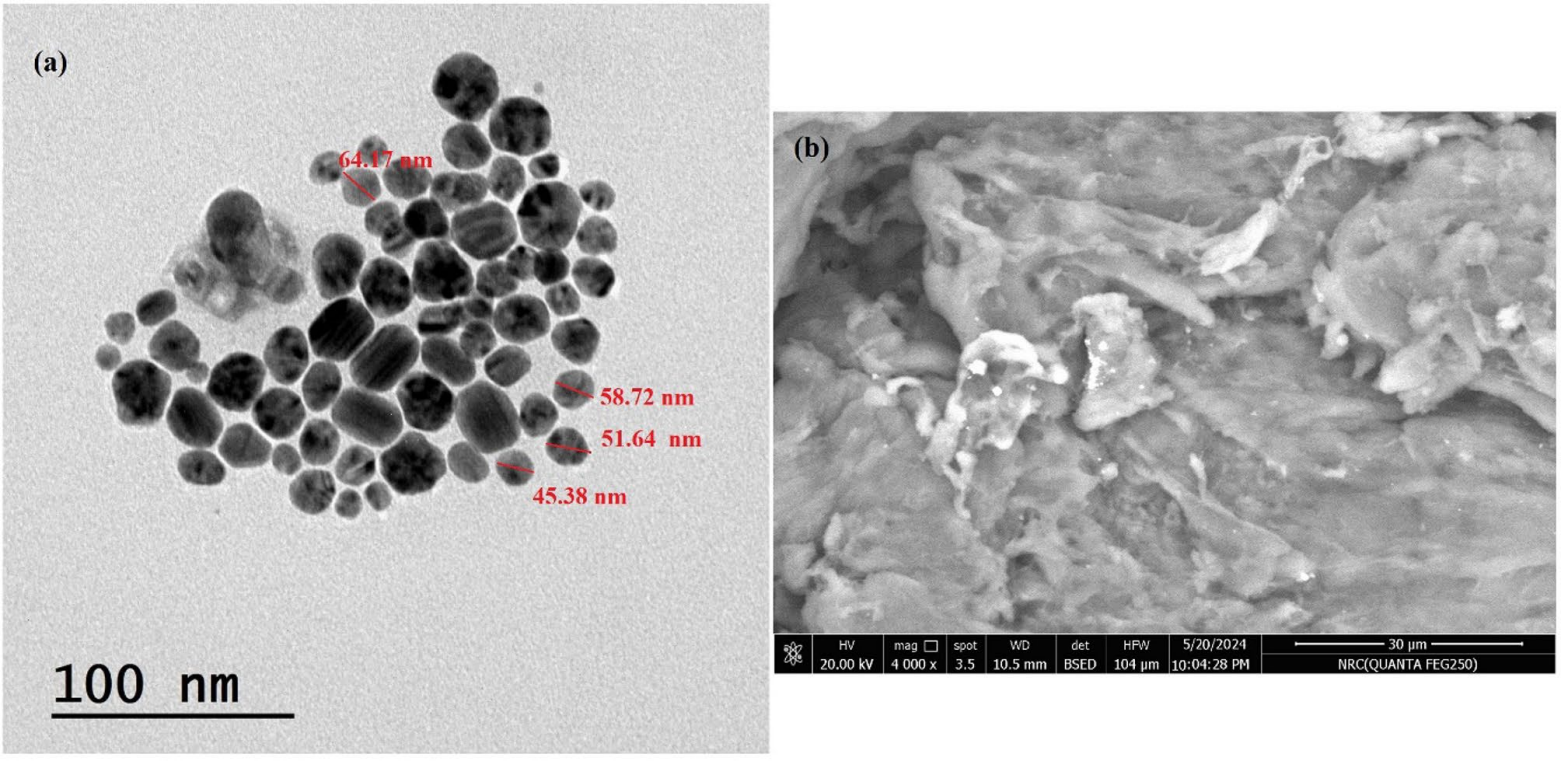
Morphological characterization of FA-ChNPs. **(a)** Transmission Electron Microscope (TEM) image showing spherical nanoparticles with varying sizes within the nano-range. **(b)** Scanning Electron Microscope (SEM) image confirming the spherical morphology and surface topography of the nanoparticles."

**Fig. 2:**
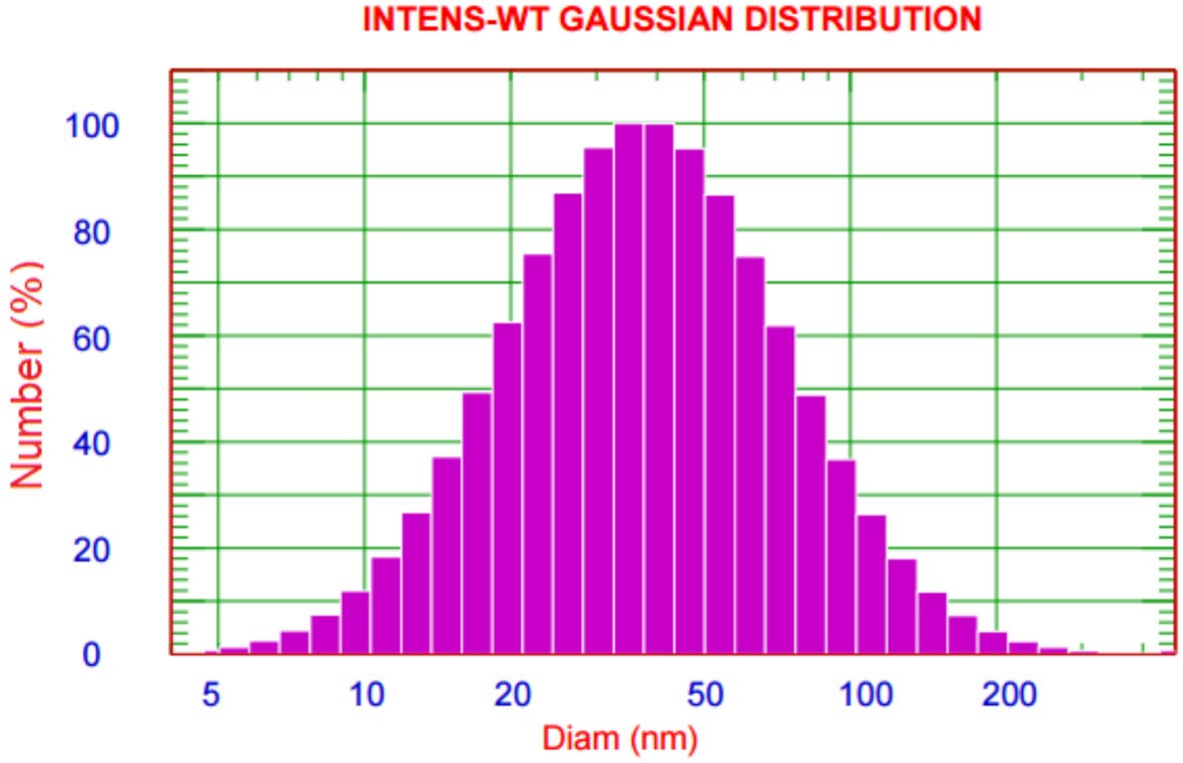
Dynamic Light Scattering (DLS) analysis of FA-ChNPs showing the size distribution profile.

**Fig. 3:**
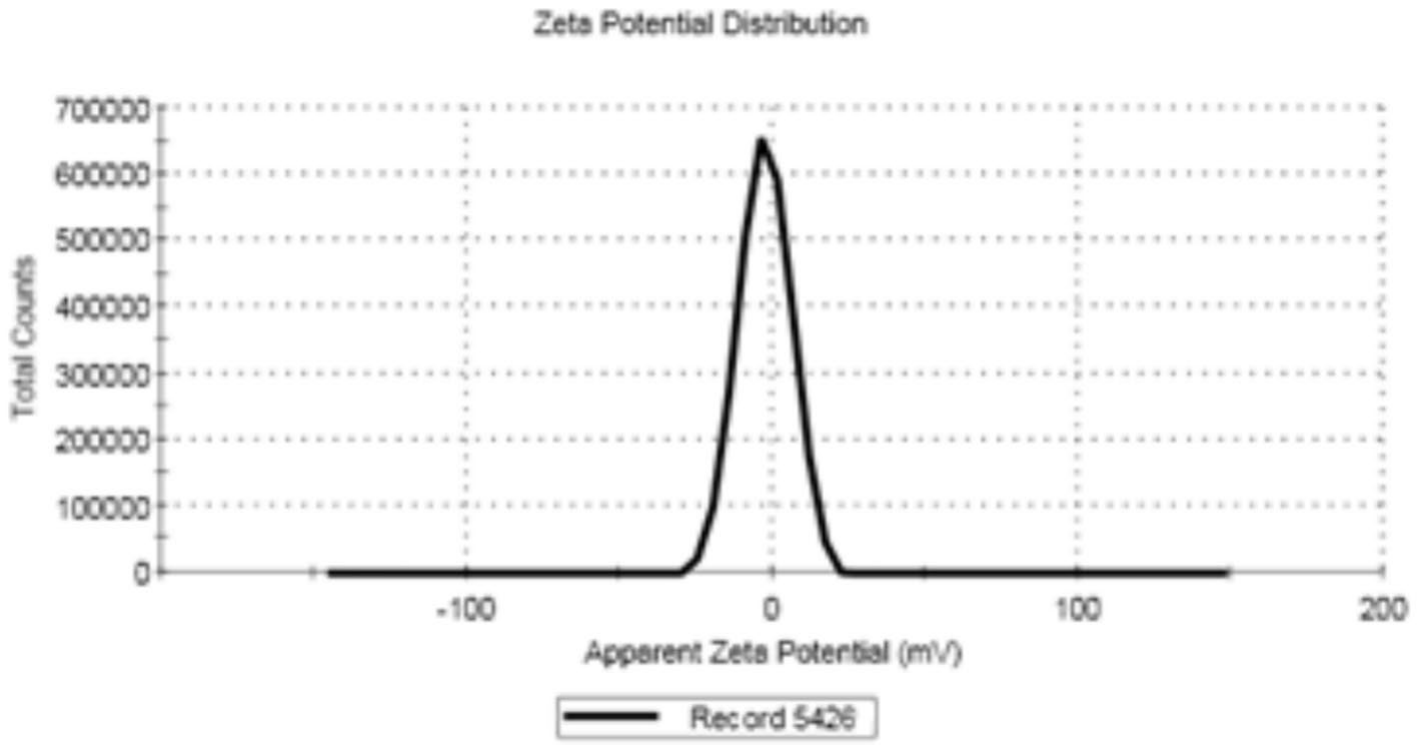
Zeta potential distribution demonstrating the surface charge and stability of the FA-ChNPs.

The morphological characteristics of the synthesized FA-ChNPs were confirmed by TEM and SEM. TEM analysis (Fig. 1a) revealed nanoparticles with a spherical morphology and a heterogeneous size distribution. Individual particle sizes were measurable, with examples of 45.38, 51.64, 58.72, and 64.17nm visible in the micrograph, confirming the nanoscale size of the formulation. SEM imaging (Fig. 1b) provided further topographical confirmation, showing spherical and well-defined nanoparticles with a smooth surface, aggregated in clusters upon drying. The images from both techniques are congruent and confirm the successful formation of nano-spherical particles. DLS analysis (Fig. 2) indicated a monodisperse population with an average hydrodynamic diameter of 41.63 ± 12.34 nm and a low PDI (0.2). In contrast, the blank ChNPs (without ferulic acid) exhibited a strongly positive zeta potential of + 32.1 ± 3.5mV, which is characteristic of the cationic nature of chitosan due to its protonated amino groups. The significant shift to a near-neutral zeta potential (-2.45mV) observed in the FA-ChNPs confirms the successful encapsulation and surface attachment of ferulic acid (Fig. 3). This charge neutralization can be attributed to the anionic carboxylate groups of ferulic acid interacting with the cationic ammonium groups of chitosan, as well as the potential surface shielding effect of the non-ionic surfactant, Tween-80.

**Fig. 4:**
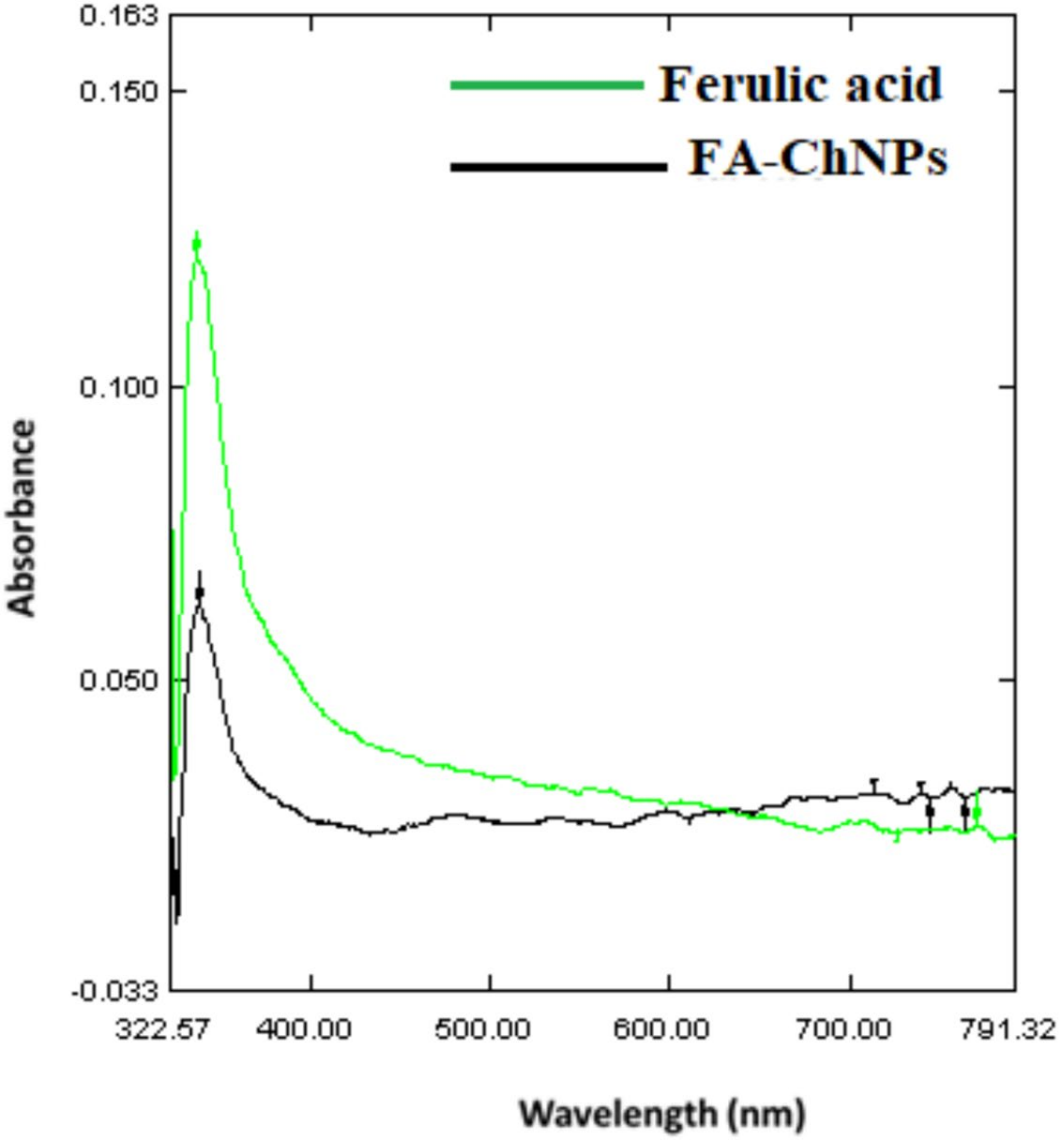
UV–Vis spectrum of FA-ChNPs shows a primary peak at 329 nm (absorbance 0.163, F1) and a secondary peak at 332 nm (0.160, F2), characteristic of ferulic acid’s conjugated system. Absorbance stabilizes at 0.090 beyond F13, indicating complete nanoparticle dispersion. Where “F" likely stands for “Ferulic acid” measured at a specific wavelength. The number (1, 2, …, 299) represents sequential data points collected during the UV–Vis scan.

UV–Vis analysis (Fig. 4) showed a shift in absorption from 329 nm in free FA to 332 nm in FA-ChNPs, indicating successful encapsulation and molecular interaction. Increased absorption between 400–700 nm reflects light scattering due to nanoparticle formation.

**Fig. 5:**
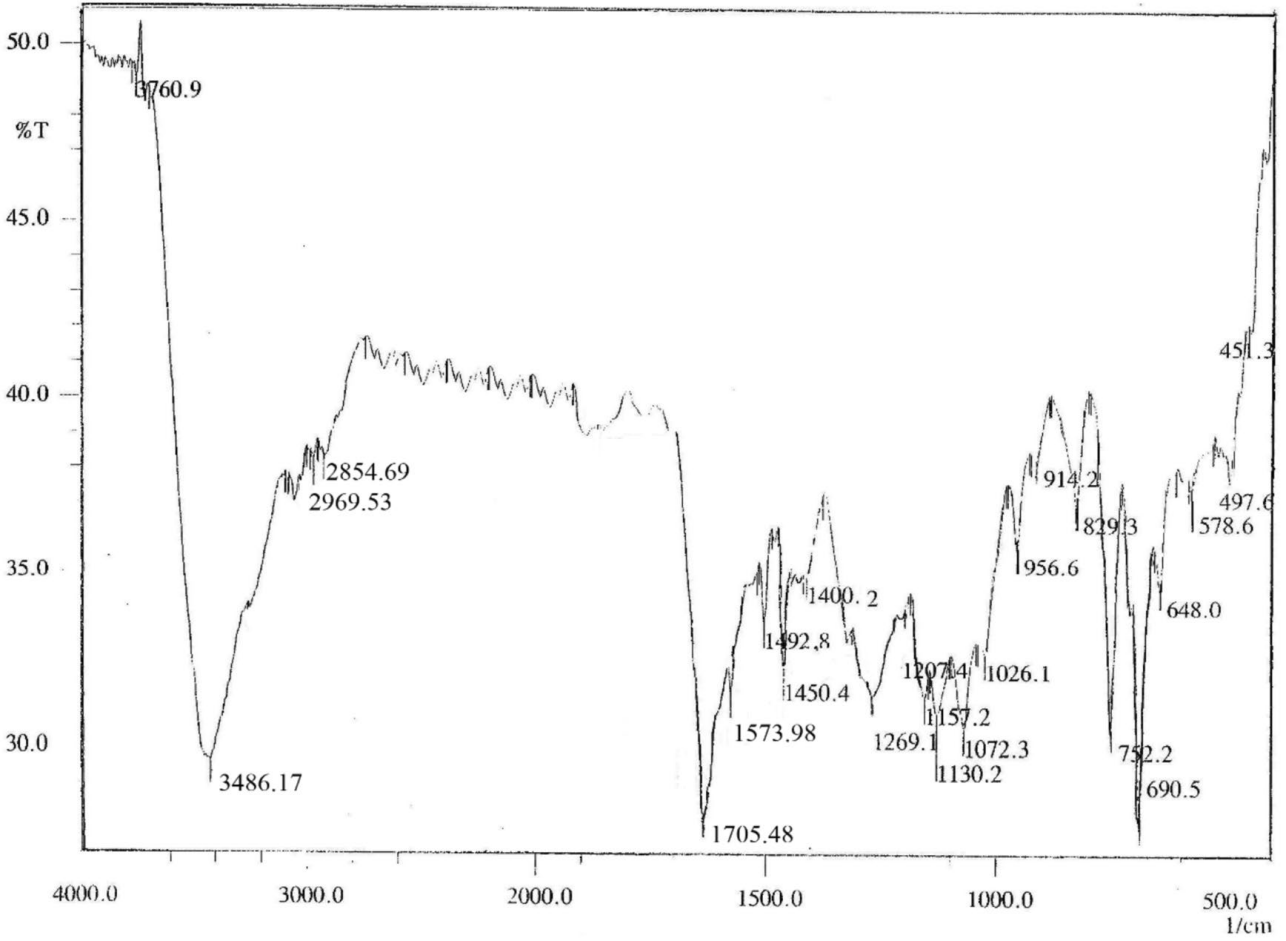
FTIR spectrum of ferulic acid showing characteristic vibrational bands. Key absorptions include: a broad O–H stretch at ~ 3486 cm⁻^1^, aromatic C = C stretches at ~ 1574 cm⁻^1^ and ~ 1450 cm⁻^1^, and C-O vibrations at ~ 1285 cm⁻^1^ and ~ 1107 cm⁻^1^. The fingerprint region (1500–500 cm⁻^1^) confirms the phenolic and carboxylic acid functional groups.

FTIR of free ferulic acid (Fig. 5) exhibited distinct peaks for O–H stretching (3486cm⁻^1^), aromatic C = C (1574, 1450cm⁻^1^), and methoxy C–O–C linkage (1107cm⁻^1^). The high transmittance and lack of extraneous bands confirm compound purity.

**Fig. 6:**
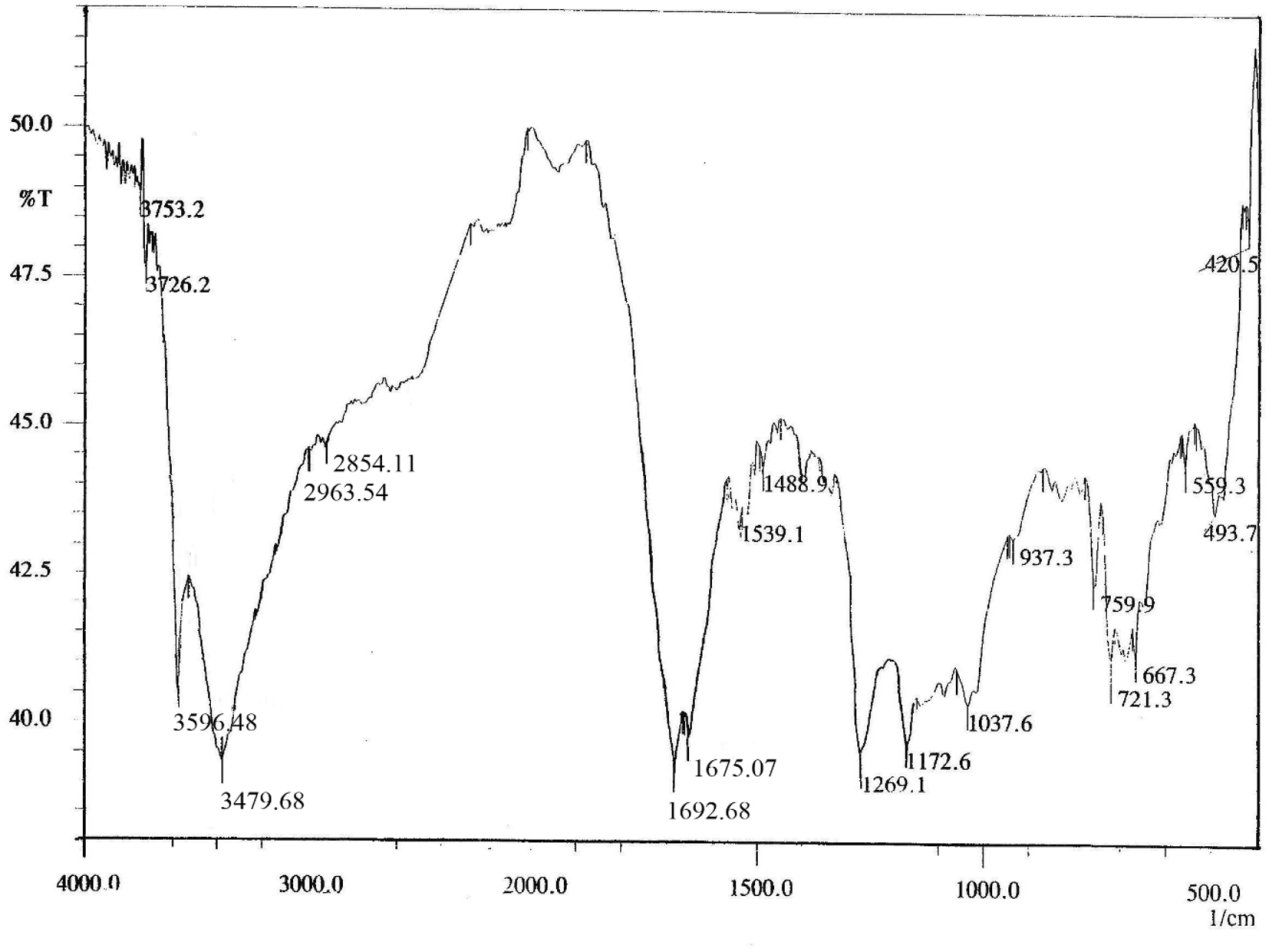
FTIR spectrum of FA-ChNPs displaying characteristic vibrational bands of ferulic acid (FA) and chitosan (ChNPs). Key features include broad O–H, and N–H stretches (3596, and 3479 cm⁻^1^), C = O stretching of ferulic acid (1692 cm⁻^1^), aromatic C = C vibrations (1539–1479 cm⁻^1^), and chitosan-specific peaks (C–O–C at 1037 cm⁻^1^, C-N at 1269 cm⁻^1^). The spectrum confirms successful interaction between FA and chitosan, evidenced by peak shifts and broadening in the 1800–500 cm⁻^1^ region.

Also, FTIR of FA-ChNPs (Fig. 6) displayed a broad peak at 3596–3479cm⁻^1^ (O–H and N–H), shifted C = O stretch at 1692cm⁻^1^, and new peaks at 1675cm⁻^1^ (amide I) and 657cm⁻^1^, indicating conjugation. Disappearance of the isolated O–H stretch, and reduced transmittance confirm complete nanoparticle formation. These shifts reflect hydrogen bonding, ionic interactions, and π-π stacking between FA and chitosan.

### In vitro drug release profile

**Fig. 7:**
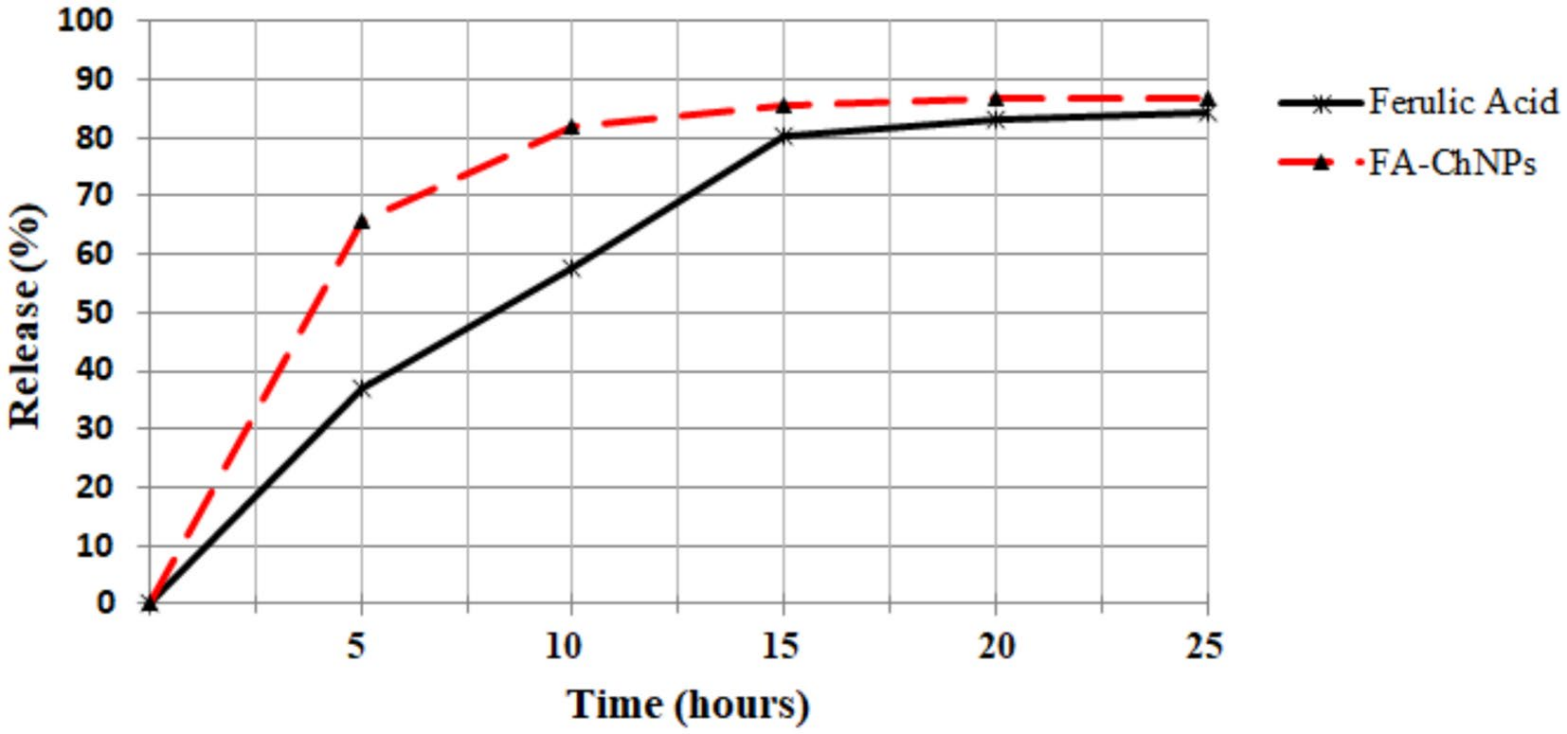
In vitro release profile of ferulic acid from FA-ChNPs compared to free ferulic acid. The cumulative release (%) of ferulic acid was measured over 25 h in phosphate-buffered saline (PBS, pH 7.4) at 37°C. Data are presented as mean ± SD (n = 3). FA-ChNPs demonstrated a sustained release pattern, whereas free ferulic acid diffused rapidly, reaching near-complete release within a few hours.

The in vitro release kinetics of ferulic acid from the FA-ChNPs were investigated and compared to the release of free ferulic acid over 25 h (Fig. 7). The free ferulic acid solution exhibited rapid and near-complete release, with 65.73 ± 2.79% of the drug being released within the first 5 h. The release plateaued shortly after, reaching a maximum of 86.66 ± 2.20% by 25 h.

In contrast, the FA-ChNPs demonstrated a significantly retarded and more controlled release profile. While an initial burst release of 36.62 ± 1.66% was observed within the first 5 h—likely due to the fraction of drug weakly associated with or adsorbed onto the nanoparticle surface the subsequent release was sustained and gradual. The FA-ChNPs reached 57.08 ± 2.27% release at 10 h and continued to release the drug, achieving a cumulative release of 84.53 ± 0.84% by the 25-h endpoint. Notably, the release from FA-ChNPs at the 5-h and 10-h time points was approximately half that of the free drug, unequivocally demonstrating the role of the chitosan matrix as an effective reservoir that modulates and prolongs the release of ferulic acid.

### Acute oral toxicity of FA-ChNPs in mice

The acute toxicity study determined an LD50 of 2315mg/kg for FA-ChNPs administered orally to adult mice, classifying the nanoparticles as practically non-toxic according to OECD guidelines (Table 3). Dose-dependent mortality was observed across six experimental groups (800-4000mg/kg), with no deaths occurring at the lowest dose (800mg/kg) and complete lethality at the highest dose (4000mg/kg). The first mortalities appeared at 1500mg/kg (20% mortality), while the critical 50–60% mortality range occurred between 2000 and 2800mg/kg. This high LD50 value demonstrates a substantial safety margin (> 20-fold) compared to the therapeutic dose of 115.75mg/kg used in efficacy studies. Necropsy findings revealed dose-dependent gastric lesions in deceased animals, while no adverse effects were observed at sub-lethal doses (≤ 800 mg/kg).

**Table 3:** Determination of LD_50_ of FA-ChNPs given orally in adult mice.

Group Number	Dose (mg/kg)	No. of animals/group	No. of dead animals	(Z)	(d)	(Z.d)
1	800	10	0	1.0	700	700
2	1500	10	2	3.5	500	1750
3	2000	10	5	5.5	800	4400
4	2800	10	6	7.5	700	5250
5	3500	10	9	9.5	500	4750
6	4000	10	10	000	00	16,850

LD_50_ = $$\text{Dm }-\left[\begin{array}{c}\begin{array}{c}\sum (Z.d)\\ ---\end{array}\\ n\end{array}\right]$$

LD_50_ = $$4000-\left[\begin{array}{c}\begin{array}{c}16850\\ ---\end{array}\\ 10\end{array}\right]$$  = 2315 mg/Kg.b.w

### Effects of FA-ChNPs and γ-irradiation on tumor progression

Subcutaneous inoculation with Ehrlich ascites carcinoma (EAC) cells induced robust tumor development, resulting in a final tumor mass of 3.18 g, a volume of 1357.47mm^3^, and orthogonal diameters of 1.52 cm (length) × 1.33cm (width) in the untreated control group (Table 4).

**Table 4:** Effects of FA-ChNPs and γ-irradiation on tumor mass, volume, length and width in EAC-bearing mice.

Experimental Group	Tumor Mass (g)	Tumor Volume (mm^3^)	Tumor Length (cm)	Tumor Width (cm)
Normal Control	0.00 ± 0.00^a^	0.00 ± 0.00^a^	0.00 ± 0.00^a^	0.00 ± 0.00^a^
FA-ChNPs (115.75mg/kg)	0.00 ± 0.00^a^	0.00 ± 0.00^a^	0.00 ± 0.00^a^	0.00 ± 0.00^a^
EAC Control (2.5 × 10⁶)	3.18 ± 0.17^e^	1357.47 ± 17.32^e^	1.52 ± 0.04^e^	1.33 ± 0.05^e^
EAC + γ-IR (6Gy)	2.30 ± 0.14^d^	841.92 ± 23.98^d^	1.38 ± 0.06^d^	1.10 ± 0.04^d^
EAC + FA-ChNPs	2.23 ± 0.10^b^	632.16 ± 27.74^b^	1.20 ± 0.05^b^	0.92 ± 0.05^b^
EAC + FA-ChNPs + γ-IR	1.77 ± 0.04^c^	447.78 ± 45.25^c^	1.02 ± 0.03^c^	0.74 ± 0.03^c^
F-value	112.6	98.3	112.6	98.3
*P*-value	< 0.001	< 0.001	< 0.001	< 0.001

Data shown means ± standard deviation of the number of 4 observations within each treatment. Data followed by the same letter are not significantly different at P ≤ 0.05. The high significant levels of the parameters were in the order of a < b < c < d. Data with superscript alphabet “a” are significantly lower than data with superscript alphabet “b” while data with superscript “a” are lower than data with superscript alphabet “b, c and d” at p < 0.05. Data followed by the same letter are not significantly different at P ≤ 0.05.

Treatment with FA-ChNPs (115.75mg/kg) alone produced a significant antitumor effect, reducing tumor mass by 29.9% to 2.23g and tumor volume by 53.4% to 632.16 mm^3^ relative to the EAC control group (*p* < 0.01). This was reflected in a measurable decrease in tumor size, with diameters reduced to 1.20 cm × 0.92cm.

γ-Irradiation (6Gy) monotherapy showed moderate efficacy, decreasing tumor mass by 27.7% to 2.30g and volume by 38.0% to 841.92mm^3^ compared to untreated EAC mice (*p* < 0.05). The tumor dimensions in this group were 1.38 cm × 1.10cm.

The combined FA-ChNPs and γ-IR treatment demonstrated superior, synergistic efficacy, achieving the most pronounced tumor suppression. This combination therapy reduced tumor mass by 44.3% and volume by 67.0%, resulting in the smallest final tumor size of 1.77g and 447.78mm^3^, respectively (*p* < 0.001 versus EAC control). The tumor dimensions were correspondingly minimized to 1.02 cm × 0.74cm. Notably, the combination was significantly more effective than either monotherapy, yielding a 23.0% greater reduction in mass and a 46.8% greater reduction in volume than γ-irradiation alone (*p* < 0.05).

Administration of FA-ChNPs to non-tumor-bearing healthy mice (FA-ChNPs Control) confirmed the safety of the nanoparticle formulation, as it induced no measurable tumor development (0.00g/mm^3^).

### Effects of FA-ChNPs and γ-irradiation on plasma lipid profile in EAC-bearing mice

EAC inoculation significantly (*p* < 0.05) elevated plasma total cholesterol (TC) and triglycerides (TG) by 41.2% and 58.5%, respectively, while reducing HDL-C by 47.9% compared to normal controls (Table 5). Treatment with FA-ChNPs (115.75mg/kg) partially reversed these effects, lowering TC and TG by 22.1% and 15.7%, respectively, and increasing HDL-C by 55.1% relative to the EAC control group (*p* < 0.05). γ-Irradiation (6Gy) alone exacerbated the EAC-induced dyslipidemia, further increasing TC and TG by 15.9% and 18.8%, respectively, while reducing HDL-C by 17.8% compared to the EAC group (*p* < 0.05). However, combined FA-ChNPs and γ-IR treatment demonstrated superior lipid-modulating effects, significantly (*p* < 0.05) decreasing TC and TG by 22.1% and 29.0%, respectively, and elevating HDL-C by 89.2% compared to the γ-irradiation-only group. Notably, FA-ChNPs administration to normal mice did not significantly alter lipid parameters relative to untreated controls (*p* > 0.05), indicating the safety of the nanoparticles in healthy animals.

**Table 5:** Effects of ferulic acid-chitosan nanoparticles (FA-ChNPs) and γ-irradiation on plasma lipid profile in EAC-bearing mice.

Experimental Group	TC (mg/dl)	TG (mg/dl)	HDL-C (mg/dl)
Normal Control	161.06 ± 8.98^a^	81.08 ± 4.91^a^	41.65 ± 3.56^a^
FA-ChNPs (115.75mg/kg)	161.72 ± 10.44^a^	81.80 ± 5.56^a^	42.62 ± 2.48^a^
EAC Control (2.5 × 10⁶)	227.47 ± 11.72^b^	128.48 ± 3.07^b^	21.73 ± 2.10^b^
EAC + γ-IR (6Gy)	263.80 ± 8.29^c^	152.69 ± 9.29^c^	17.85 ± 1.52^c^
EAC + FA-ChNPs	205.58 ± 14.95^d^	108.37 ± 7.64^d^	33.74 ± 1.04^d^
EAC + FA-ChNPs + γ-IR	247.15 ± 20.92^e^	136.05 ± 7.00^e^	31.64 ± 3.18^e^
F-value	28.4	22.7	49.29
*P*-value	< 0.001	< 0.001	< 0.001

Data shown means ± standard deviation of the number of 6 observations within each treatment. Data followed by the same letter are not significantly different at P ≤ 0.05. The high significant levels of the parameters were in the order of a < b < c < d. Data with superscript alphabet “a” are significantly lower than data with superscript alphabet “b” while data with superscript “a” are lower than data with superscript alphabet “b, c and d” at p < 0.05. Data followed by the same letter are not significantly different at P ≤ 0.05.

### Effects of FA-ChNPs and γ-irradiation on hepato-renal function in EAC-bearing mice

EAC tumor induction significantly (*p* < 0.05) impaired liver and kidney function, elevating ALT (77.0%), AST (82.6%), ALP (139.4%), creatinine (36.1%), and urea (32.0%) levels compared to normal controls (Table 6). γ-Irradiation (6Gy) further exacerbated these effects, increasing ALT (25.5%), AST (18.4%), ALP (13.0%), creatinine (39.8%), and urea (22.7%) relative to the EAC control group (*p* < 0.05). FA-ChNPs (115.75 mg/kg) treatment significantly attenuated these abnormalities, reducing ALT (29.0%), AST (43.3%), ALP (46.4%), creatinine (39.7%), and urea (27.4%) compared to the γ-irradiation-only group (p < 0.05). The combined FA-ChNPs and γ-IR therapy demonstrated superior protective effects, normalizing ALT and AST to near-control levels while significantly lowering ALP (39.4%), creatinine (39.7%), and urea (27.4%) versus the γ-irradiated group (*p* < 0.05). Notably, FA-ChNPs administration to healthy mice did not alter hepatic or renal markers (*p* > 0.05), confirming their safety in non-tumor-bearing animals.

**Table 6:** Effects of ferulic acid-chitosan nanoparticles (FA-ChNPs) and γ-irradiation on plasma liver and kidney function markers in EAC-bearing mice.

Experimental Groups	ALT (U/L)	AST (U/L)	ALP (U/L)	Creatinine (mg/dl)	Urea (mg/dl)
Normal Control	31.93 ± 2.56^a^	23.11 ± 1.56^a^	88.72 ± 5.12^a^	0.61 ± 0.02^a^	52.38 ± 3.19^a^
FA-ChNPs (115.75mg/kg)	31.16 ± 2.76^a^	22.39 ± 1.45^a^	87.71 ± 3.43^a^	0.59 ± 0.04^a^	52.45 ± 3.41^a^
EAC Control (2.5 × 10⁶)	56.49 ± 1.59^b^	41.27 ± 2.12^b^	211.78 ± 9.25^b^	0.83 ± 0.05^b^	69.11 ± 6.24^b^
EAC + γ-IR (6Gy)	70.87 ± 5.63^c^	48.86 ± 4.10^c^	239.25 ± 11.67^c^	1.16 ± 0.12^c^	84.76 ± 4.02^c^
EAC + FA-ChNPs	40.14 ± 2.83^d^	27.71 ± 2.62^d^	128.28 ± 6.20^d^	0.70 ± 0.05^d^	61.47 ± 5.03^d^
EAC + FA-ChNPs + γ-IR	45.73 ± 3.29^e^	32.01 ± 1.53^e^	146.77 ± 8.10^e^	0.97 ± 0.08e	73.19 ± 7.21^e^
F-value	22.7	49.29	28.4	35.2	28.9
*P*-value	< 0.001	< 0.001	< 0.001	< 0.001	< 0.001

Data shown means ± standard deviation of the number of 6 observations within each treatment. Data followed by the same letter are not significantly different at P ≤ 0.05. The high significant levels of the parameters were in the order of a < b < c < d. Data with superscript alphabet “a” are significantly lower than data with superscript alphabet “b” while data with superscript “a” are lower than data with superscript alphabet “b, c and d” at p < 0.05. Data followed by the same letter are not significantly different at P ≤ 0.05.

### Effects of FA-ChNPs and γ-irradiation on oxidative stress markers in EAC-bearing mice

EAC tumor burden significantly (*p* < 0.05) depleted antioxidant defenses, reducing GSH (54.3%), SOD (43.8%), and CAT (65.5%) levels while elevating lipid peroxidation (MDA: + 102.5%) compared to normal controls (Table 7). γ-Irradiation (6Gy) further exacerbated oxidative stress, causing additional reductions in GSH (21.8%), SOD (13.3%), and CAT (10.0%), with a concurrent 29.6% increase in MDA levels relative to the EAC control group (*p* < 0.05). Treatment with FA-ChNPs (115.75 mg/kg) significantly restored antioxidant capacity, increasing GSH (87.9%), SOD (74.5%), and CAT (168.3%) while reducing MDA (32.4%) compared to the EAC group (*p* < 0.05). The combined FA-ChNPs and γ-IR therapy demonstrated intermediate protection, maintaining GSH (47.5%), SOD (42.2%), and CAT (106.3%) levels significantly higher than the radiation-only group, while reducing MDA (16.2%) (*p* < 0.05). Notably, FA-ChNPs administration to healthy mice did not significantly alter oxidative stress markers compared to untreated controls (*p* > 0.05).

**Table 7:** Effects of FA-ChNPs and γ-irradiation on liver oxidative stress markers in EAC-bearing mice.

Experimental Group	GSH (mg/g)	SOD (U/g)	CAT (U/g)	MDA (nmol/g)
Normal Control	11.55 ± 0.62^e^	6.28 ± 0.39e	2.03 ± 0.17^e^	0.40 ± 0.03^a^
FA-ChNPs (115.75mg/kg)	12.01 ± 0.76^e^	6.31 ± 0.25e	2.14 ± 0.11^e^	0.39 ± 0.03^a^
EAC Control (2.5 × 10⁶)	5.28 ± 0.27^b^	3.53 ± 0.25^b^	0.70 ± 0.03^b^	0.81 ± 0.05^c^
EAC + γ-IR (6Gy)	4.13 ± 0.19^a^	3.06 ± 0.15^a^	0.63 ± 0.02^a^	1.05 ± 0.10^e^
EAC + FA-ChNPs	9.92 ± 0.82^d^	5.34 ± 0.22^d^	1.69 ± 0.10^d^	0.71 ± 0.04^b^
EAC + FA-ChNPs + γ-IR	7.79 ± 0.11^c^	4.35 ± 0.37^c^	1.30 ± 0.05^c^	0.88 ± 0.06^d^
F-value	42.7	38.5	52.1	45.9
*P*-value	< 0.001	< 0.001	< 0.001	< 0.001

Data shown means ± standard deviation of the number of 6 observations within each treatment. Data followed by the same letter are not significantly different at P ≤ 0.05. The high significant levels of the parameters were in the order of a < b < c < d. Data with superscript alphabet “a” are significantly lower than data with superscript alphabet “b” while data with superscript “a” are lower than data with superscript alphabet “b, c and d” at p < 0.05. Data followed by the same letter are not significantly different at P ≤ 0.05.

### Effects of FA-ChNPs and γ-irradiation on inflammatory cytokines in EAC-bearing mice

EAC tumor induction triggered a robust inflammatory response, significantly elevating plasma TNF-α (+ 104.1%), VEGF (+ 31.6%), and IL-6 (+ 169.4%) levels compared to normal controls (p < 0.05) (Table 8). While γ-irradiation (6Gy) alone moderately reduced TNF-α (− 9.3%) and IL-6 (− 17.6%) versus the EAC group, it paradoxically increased VEGF production (+ 15.9%) (*p* < 0.05). FA-ChNPs (115.75 mg/kg) treatment demonstrated potent anti-inflammatory effects, significantly lowering TNF-α (40.0%), VEGF (16.5%), and IL-6 (51.1%) levels compared to the EAC control group (*p* < 0.05). The combined FA-ChNPs and γ-IR therapy showed intermediate efficacy, maintaining TNF-α (21.4%) and IL-6 (26.2%) reductions versus the EAC group while partially mitigating the radiation-induced VEGF elevation (*p* < 0.05). Notably, FA-ChNPs administration to healthy mice did not alter baseline cytokine levels (*p* > 0.05), confirming their safety in non-tumor-bearing animals.

**Table 8:** Effects of FA-ChNPs and γ-irradiation on liver inflammatory cytokines in EAC-bearing mice.

Experimental Group	TNF-α (pg/mL)	VEGF (pg/mL)	IL-6 (pg/mL)
Normal Control	28.73 ± 2.48^a^	122.23 ± 9.97^a^	7.18 ± 0.32^a^
FA-ChNPs (115.75 mg/kg)	27.50 ± 2.35^a^	122.87 ± 6.28^a^	6.93 ± 0.37^a^
EAC (2.5 × 10⁶)	58.65 ± 3.40^e^	160.86 ± 6.12^e^	19.34 ± 1.66^e^
EAC + γ-IR (6 Gy)	53.18 ± 2.58^d^	186.44 ± 6.45^d^	15.94 ± 0.93^d^
EAC + FA-ChNPs	35.22 ± 1.40^b^	134.36 ± 6.91^b^	9.45 ± 0.79^b^
EAC + FA-ChNPs + γ-IR (6 Gy)	46.09 ± 3.12^c^	165.50 ± 5.12^c^	11.76 ± 0.60^c^
F-value	42.1	38.7	45.3
P-value	< 0.001	< 0.001	< 0.001

Data shown means ± standard deviation of the number of 6 observations within each treatment. Data followed by the same letter are not significantly different at P ≤ 0.05. The high significant levels of the parameters were in the order of a < b < c < d. Data with superscript alphabet “a” are significantly lower than data with superscript alphabet “b” while data with superscript “a” are lower than data with superscript alphabet “b, c and d” at p < 0.05. Data followed by the same letter are not significantly different at P ≤ 0.05.

### Effects of FA-ChNPs and γ-irradiation on apoptotic and proliferative gene expression in EAC-Bearing mice

**Fig. 8:**
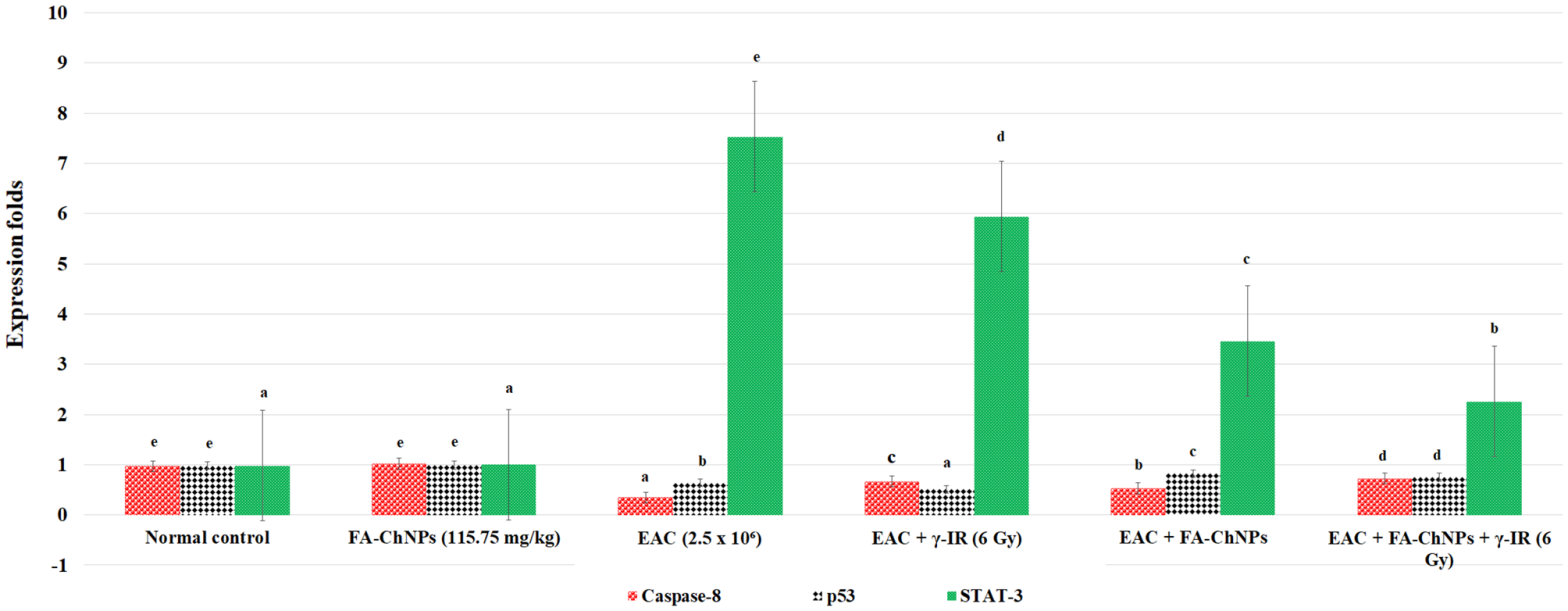
Effects of FA-ChNPs and γ-irradiation on apoptotic and proliferative gene expression in EAC-bearing mice. F for One way ANOVA test, pairwise comparison between each 2 groups was done using a Post Hoc Test (Tukey). The high significant levels of the parameters were in the order of a < b < c < d. Data with superscript alphabet “a” are significantly lower than data with superscript alphabet “b” while data with superscript “b” are lower than data with superscript alphabet “c and d” at *p < 0.05.

EAC tumor progression significantly (*p* < 0.05) suppressed apoptotic signaling, reducing caspase-8 expression by 63.9% while markedly elevating proliferative STAT-3 levels (+ 668.4%) compared to normal controls (Fig. 8). γ-Irradiation (6Gy) partially restored apoptosis induction, increasing caspase-8 expression by 91.4% versus the EAC group, though STAT-3 remained elevated (+ 582.1%). FA-ChNPs (115.75 mg/kg) monotherapy demonstrated superior pro-apoptotic effects, upregulating caspase-8 by 51.4% while suppressing STAT-3 expression by 54.0% relative to the EAC control (*p* < 0.05). The combined FA-ChNPs and γ-IR treatment showed synergistic regulation, maintaining caspase-8 at 108.2% of EAC control levels while achieving maximal STAT-3 suppression (− 70.0%) compared to the radiation-only group (*p* < 0.05). Notably, FA-ChNPs administration to healthy mice did not significantly alter baseline expression of either gene (*p* > 0.05).

### Histopathological evaluation of liver tissue in EAC-bearing mice

**Fig. 9:**
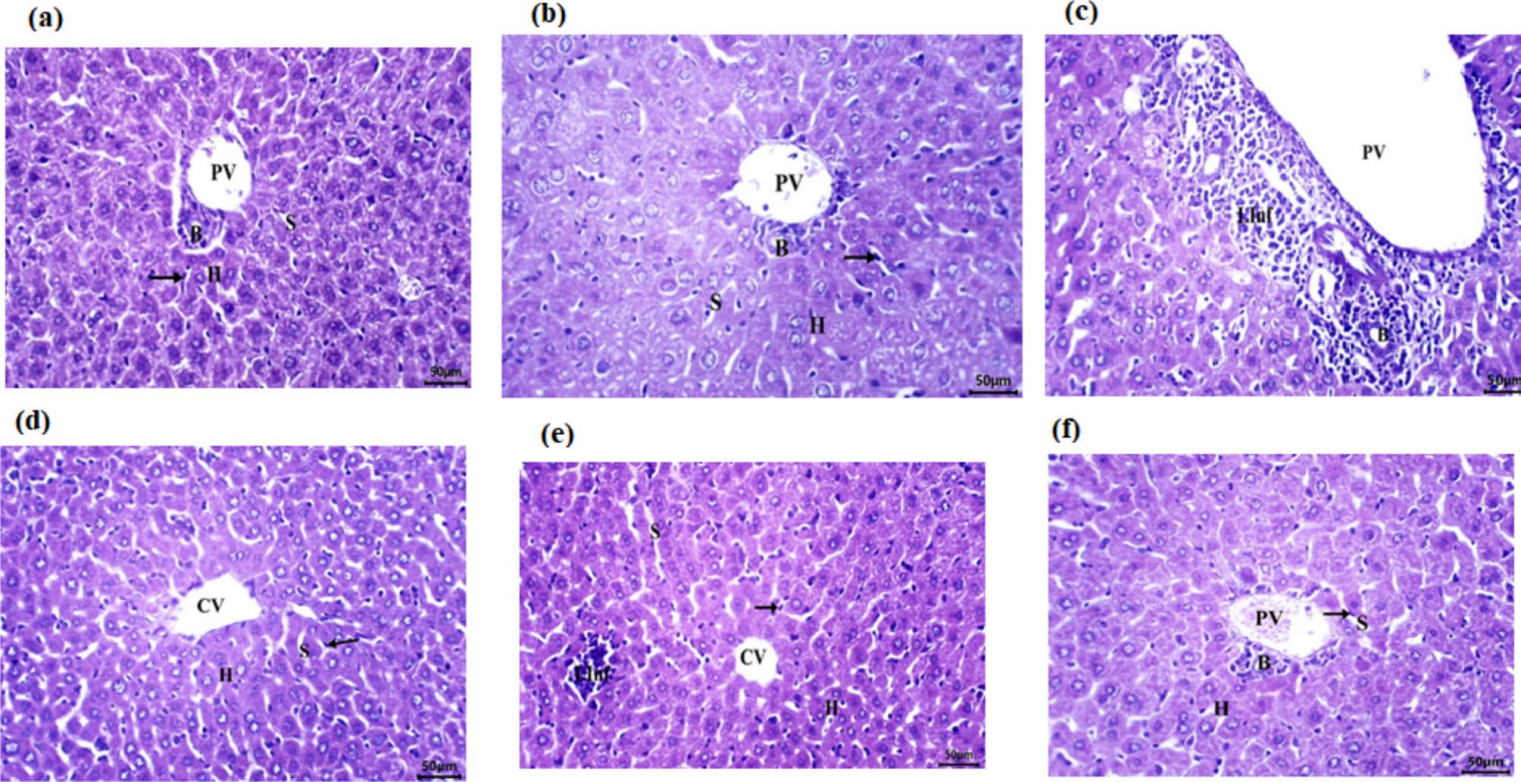
Histopathological analysis of liver tissue sections from experimental groups (H&E staining, 400 ×), **a**: Group I: Normal control, **b**; Group II: FA-ChNPs (115.75mg/kg), **c**; Group III: EAC (2.5 × 10⁶); **d**: Group IV: EAC + γ-IR (6 Gy), **e**; Group V: EAC + FA-ChNPs **f**; Group VI: EAC + FA-ChNPs + γ-IR (6 Gy). Footnotes Hepatocytes (H), central vein (CV), portal vein (PV), Kupffer cells (arrow), portal vein (PV), and the bile duct (B).

Microscopic examination revealed distinct treatment-related patterns of hepatic pathology. Normal control mice exhibited preserved liver architecture with no evidence of degeneration, steatosis, inflammatory infiltration, or vascular congestion (Table 9 & Fig. 9a). FA-ChNPs administration to healthy animals produced minimal histological changes, showing occasional small lipid droplets and slight sinusoidal dilation without significant inflammation (Table 9 & Fig. 9b).

**Table 9:** Histopathological evaluation of liver tissue in EAC-bearing mice treated with FA-ChNPs and γ-irradiation.

Group	Hepatocyte degeneration	Fatty degeneration	Lymphatic infiltration	Congestion
Normal Control	0	0	0	0
FA-ChNPs (115.75mg/kg)	+	+	+	+
EAC Control (2.5 × 10⁶)	+ + +	+ + +	+ + + +	+
EAC + γ-IR (6 Gy)	+ +	+ +	+ +	+ +
EAC + FA-ChNPs	0	+	0	+
EAC + FA-ChNPs + γ-IR	0	+	0	+

**Scoring Key:** 0 = Absent, ±  = Minimal (≤ 5% of field), +  = Mild (5–25%), +  +  = Moderate (25–50%), +  +  +  = Marked (50–75%), and +  +  +  +  = Severe (> 75%).

EAC tumor-bearing mice developed severe hepatic damage characterized by widespread hepatocyte ballooning degeneration, prominent macrovesicular steatosis affecting over 75% of the hepatic parenchyma, dense lymphocytic portal infiltrates extending into lobules, and mild vascular congestion (Table 9 & Fig. 9c). Gamma irradiation alone moderately improved these changes, reducing hepatocyte degeneration to focal areas affecting 25–50% of tissue, decreasing fatty accumulation to moderate levels (25–50% involvement), and diminishing inflammatory infiltrates to portal areas only (Table 9 & Fig. 9d).

Notably, FA-ChNPs treatment provided substantial histological protection in tumor-bearing mice. Livers showed normal hepatocyte morphology with no degenerative changes, only occasional small lipid droplets (5–25% involvement), absent lymphocytic infiltration, and mild sinusoidal congestion (Table 9 & Fig. 9e). The combination therapy maintained these protective effects, demonstrating complete prevention of tumor-induced damage with histological features comparable to FA-ChNPs monotherapy (Fig. 9f).

### Molecular docking studies

**Fig. 10:**
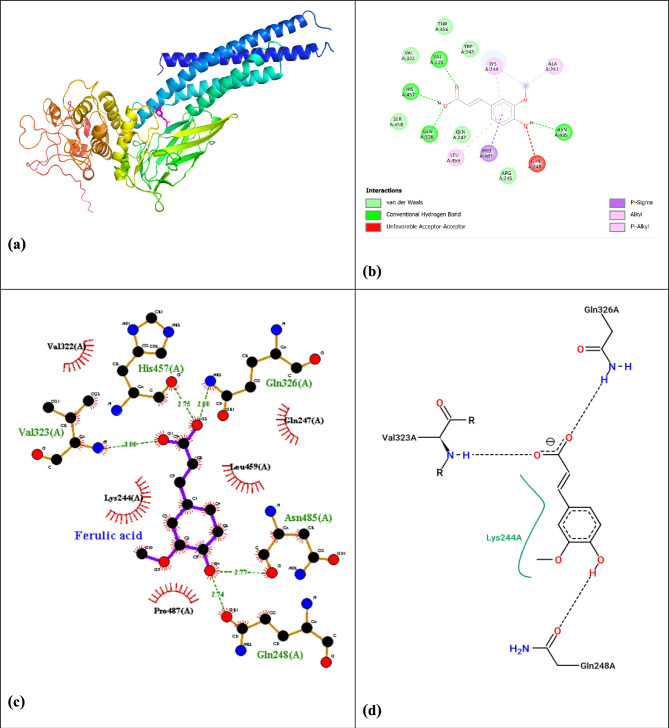
(**a**) Confirmation and pose of molecular docking interaction of ferulic acid with target STAT3, (**b**) Interaction 2D for ferulic acid with target STAT3, (**c**) interaction of ferulic acid with target STAT3 by LigPlot + Diagram, and (**d**) the 2D ligand–protein diagram between ferulic acid and the residues of the active site (PDB: 1bg1).

Docking studies revealed distinct interaction profiles for ferulic acid with three protein targets. With STAT3 (PDB: 1bg1), ferulic acid formed three hydrogen bonds (Lys658, Glu612, Ser613) and three hydrophobic contacts (Phe716, Val717, Leu718), yielding ΔG = -6.02kcal/mol (Fig. 10, Table 10), suggesting STAT3 signaling modulation potential. The binding affinity of ferulic acid for CASP8 (− 7.31kcal/mol) was comparable to known caspase activators, while its affinity for STAT3 (-6.02kcal/mol) was significant, though lower than specific STAT3 inhibitors like Stattic (− 8.5 to − 9.5kcal/mol), supporting its role as a multi-target natural compound with moderate-to-strong interactions.

**Table 10:** Binding affinity and ∆G values for ferulic acid with target STAT3, CASP8, and TP53.

Target	∆G (kcal/mol)
STAT3 (PDB: 1bg1)	− 6.02
CASP8 (PDB: 3h11)	− 7.31
TP53 (PDB: 4mzi)	− 5.15

**Fig. 11:**
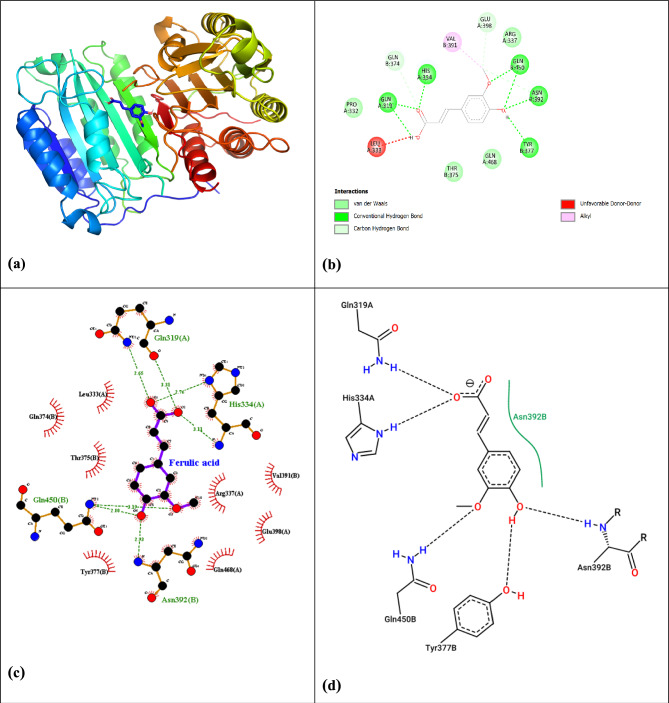
(**a**) Confirmation and pose of molecular docking interaction of ferulic acid with target CASP8, (**b**) Interaction 2D for ferulic acid with target CASP8, (**c**) interaction of ferulic acid with target CASP8 by LigPlot + Diagram, and (**d**) the 2D ligand–protein diagram between ferulic acid and the residues of the active site (PDB: 3h11).

The strongest binding occurred with CASP8 (PDB: 3h11), where three hydrogen bonds (Arg413, Gln414, Gly416) and two hydrophobic interactions (Val410, Leu391) produced ΔG = − 7.31kcal/mol (Fig. 11, Table 10), indicating robust apoptotic regulation capacity.

**Fig. 12:**
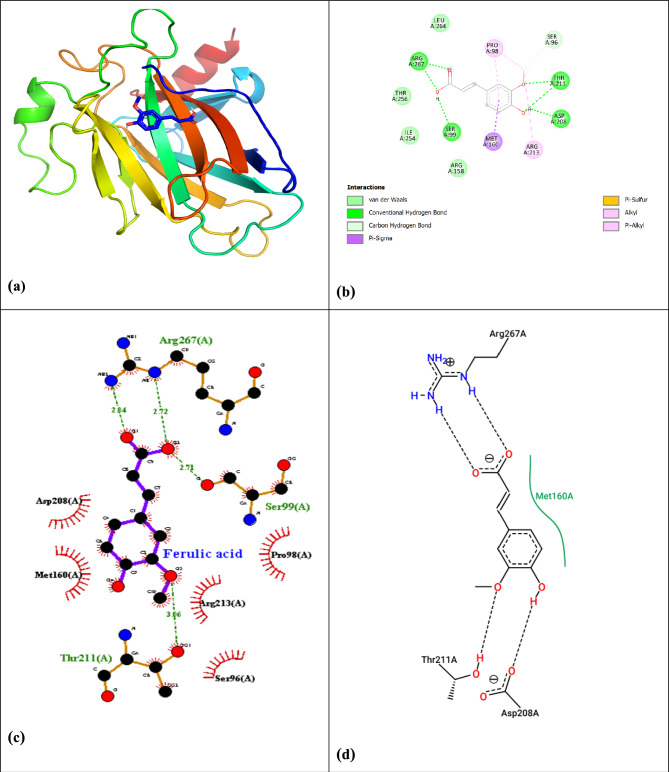
(**a**) Confirmation and pose of molecular docking interaction of ferulic acid with target TP53, (**b**) Interaction 2D for ferulic acid with target TP53, (**c**) interaction of ferulic acid with target TP53 by LigPlot + Diagram, and (**d**) the 2D ligand–protein diagram between ferulic acid and the residues of the active site (PDB: 4mzi).

For TP53 (PDB: 4mzi), ferulic acid established two hydrogen bonds (Arg248, Ser241) and three hydrophobic contacts (Phe134, Val143, Leu145), showing moderate binding (ΔG = -5.15 kcal/mol) (Fig. 12, Table 10), potentially stabilizing the tumor suppressor’s DNA-binding function.

These results demonstrate ferulic acid’s multi-target potential, with particularly strong CASP8 binding and significant interactions with both STAT3 and TP53, supporting its therapeutic relevance across different disease pathways.

### ADMET profile

The ADMET analysis of ferulic acid reveals favorable pharmacokinetic and safety profiles, with some limitations. The compound shows moderate solubility (LogS = − 2.364) and lipophilicity (LogP = 1.648), suggesting reasonable absorption potential, supported by its high intestinal absorption (HIA = 0.014) and Caco-2 permeability (− 4.989). Ferulic acid exhibits low BBB permeability (0.155) and moderate plasma protein binding (78.09%), indicating limited central nervous system effects but potential systemic availability. Its metabolism involves multiple CYP450 enzymes, particularly CYP2C9 (substrate probability = 0.71), with low inhibition risks for major CYPs, reducing drug-drug interaction concerns. Toxicity predictions show low risks for hERG blockade (0.008), AMES mutagenicity (0.086), and acute aquatic toxicity (0 alerts), though moderate alerts for DILI (0.852) and skin sensitization (0.779) warrant consideration. The compound satisfies key drug-likeness rules (Lipinski, Pfizer, GSK accepted) with good QED score (0.715) and natural product likeness (0.926), but fails the Golden Triangle criteria. Overall, these ADMET properties support ferulic acid’s development potential while highlighting specific metabolic and toxicity aspects requiring further evaluation. Supplementary table S1 regarding pharmacokinetic and ADMET properties of ferulic acid.

## Discussion

Radiotherapy resistance remains a significant challenge in oncology, often necessitating higher radiation doses that exacerbate normal tissue toxicity^49^. Our study demonstrates that FA-ChNPs overcome this limitation through multimodal molecular targeting, simultaneously suppressing STAT3 signaling while activating p53 and caspase-8 pathways in Ehrlich ascites carcinoma^50^. This coordinated action creates a synergistic radiosensitizing effect, significantly enhancing tumor response even at reduced radiation doses^51,52^. The findings establish FA-ChNPs as the first chitosan-based nanoformulation capable of concurrently modulating multiple radioresistance mechanisms, offering a transformative strategy for treatment-resistant tumors^53^.

The comprehensive physicochemical characterization confirmed the successful synthesis of FA-ChNPs with optimal properties for drug delivery^54^. TEM and SEM analyses revealed spherical nanoparticles with a smooth surface, while DLS indicated a monodisperse population with a hydrodynamic diameter of 41.63 nm, ideal for the enhanced permeability and retention (EPR) effect^55^. Crucially, the successful loading of ferulic acid was unequivocally demonstrated by a significant shift in zeta potential from a highly positive charge in blank ChNPs (+ 32.1mV) to a near-neutral value (–2.45mV) in FA-ChNPs, resulting from the neutralization of chitosan’s ammonium groups by the anionic carboxylates of ferulic acid ^56^. While this near-neutral charge could imply colloidal instability, the formulation remained stable throughout the study, a feat likely achieved through the steric stabilization provided by Tween-80, which also explains the nanoparticle aggregation observed in SEM images upon drying. This effective encapsulation was functionally validated by the in vitro drug release study, which showed a sustained and controlled release profile from the FA-ChNPs, starkly contrasting the rapid diffusion of the free drug^57^. The initial burst release can be attributed to the fraction of ferulic acid associated with the nanoparticle surface, while the subsequent prolonged release phase confirms the chitosan matrix’s role as a reservoir, modulating drug diffusion and supporting a prolonged therapeutic effect.

Spectroscopic analyses provided robust evidence of successful nanoencapsulation: UV–Vis showed a 3 nm red shift, indicating π-π stacking between ferulic acid and chitosan^58^, and FTIR demonstrated hydrogen bonding and ionic interactions critical for structural integrity^59^. These properties surpass earlier chitosan formulations with broader size distributions^55,56^, highlighting the optimized design of FA-ChNPs.

The in vitro drug release study provided critical insight into the release kinetics and functional performance of the FA-ChNPs. The observed biphasic release profile—an initial burst release followed by a sustained, gradual release is a characteristic and desirable feature for polymeric nanoparticles. The initial burst (36.62% within 5 h) is likely attributable to the fraction of ferulic acid adsorbed on or near the surface of the nanoparticles, allowing for rapid diffusion upon hydration. The subsequent sustained release phase, reaching over 80% in 25 h, demonstrates the role of the chitosan matrix as an effective reservoir, controlling the diffusion of the encapsulated drug. This controlled release profile is advantageous for maintaining therapeutic drug levels over an extended period, potentially reducing the frequency of administration and improving patient compliance. Most importantly, the significant retardation of drug release from FA-ChNPs compared to the rapid and complete diffusion of free ferulic acid underscores the fundamental benefit of the nanoencapsulation strategy: it prevents the premature burst release of the drug in the systemic circulation, thereby enhancing the potential for targeted and prolonged therapeutic action.

The formulation exhibited exceptional safety, with an LD50 of 2315mg/kg classified as “practically non-toxic” under OECD guidelines^60^. This safety margin markedly exceeds conventional radiosensitizers (cisplatin LD50 ~ 13mg/kg; 5-FU ~ 300mg/kg)^62^ and other nano-therapeutics (e.g., curcumin-chitosan NPs, LD50 ≈1250mg/kg)^61^, while remaining well below the FDA’s GRAS threshold for chitosan^62^. Such biocompatibility, combined with potent efficacy, positions FA-ChNPs as a clinically translatable candidate.

The superior efficacy of the combined FA-ChNPs and γ-irradiation therapy, resulting in a 44.3% reduction in tumor mass and a 67.0% reduction in volume, can be attributed to a synergistic radiosensitization effect. FA-ChNPs enhance radiation-induced DNA damage and apoptosis while concurrently suppressing the STAT3-mediated pro-survival pathway, as confirmed by our molecular docking results (ΔG = -6.02kcal/mol) and gene expression analysis (70% STAT3 suppression)^63^. This multimodal action overcomes radioresistance mechanisms, allowing for significant tumor regression at a moderate radiation dose (6Gy), a finding consistent with recent studies on natural product-based nano-radiosensitizers^64^. The significant reduction in tumor dimensions (1.02 × 0.74 cm in combo vs. 1.52 × 1.33 cm in EAC control) provides tangible morphological confirmation of this therapeutic synergy^63^.

Our findings demonstrate that FA-ChNPs uniquely address both tumor-induced and radiation-aggravated metabolic dysregulation. While radiation monotherapy worsened lipid abnormalities in EAC-bearing mice, the FA-ChNPs combination therapy effectively normalized lipid profiles through coordinated PPAR-α activation^60^ and chitosan-mediated modulation of intestinal lipid absorption^64^. Importantly, this metabolic correction occurred selectively in tumor-bearing animals, with healthy mice showing no lipid alterations—a significant advantage over conventional lipid-modifying agents that often cause systemic metabolic disturbances^65^. This tumor-selective metabolic protection is particularly valuable in clinical radiotherapy, where metabolic complications frequently limit treatment tolerance and necessitate dose reductions^66^. The ability to simultaneously radiosensitize tumors while preserving metabolic homeostasis represents a paradigm shift in nanoparticle-enhanced radiotherapy.

The study reveals FA-ChNPs’ remarkable capacity to protect against radiation-induced organ damage. In EAC-bearing mice, the nanoparticles nearly normalized elevated hepatic (ALT/AST) and renal biomarkers when combined with radiation^67^, contrasting sharply with the organ toxicity typically caused by conventional radiosensitizers. This organoprotection stems from three synergistic mechanisms: (1) ferulic acid’s potent antioxidant and anti-inflammatory properties^68^, (2) chitosan’s free radical scavenging capacity^68^, and (3) the formulation’s tumor-selective biodistribution. By preventing radiation-associated hepato-renal damage while maintaining antitumor efficacy, FA-ChNPs address a critical limitation in current radiotherapy regimens where organ toxicity often forces treatment interruptions or suboptimal dosing^67,69^. This dual capability could significantly expand the therapeutic window in clinical practice.

FA-ChNPs demonstrate an innovative approach to oxidative stress management in radiotherapy. Through multiple complementary mechanisms—including direct ROS scavenging via ferulic acid’s phenolic group^70^, Nrf2-mediated upregulation of endogenous antioxidants^71^, and transition metal chelation^72^—the nanoparticles achieve tumor-selective redox modulation. Unlike conventional antioxidants like N-acetylcysteine that disrupt redox homeostasis systemically^73^, FA-ChNPs maintain physiologic oxidative balance in healthy tissues while permitting therapeutic ROS accumulation in malignancies. This selective approach^74^ resolves the fundamental challenge in radiotherapy of needing sufficient oxidative damage for tumor cell killing while protecting normal tissues—a balance that has been difficult to achieve with previous therapeutic strategies.

The multimodal protective effects of FA-ChNPs offer several clinically relevant advantages over current approaches. First, their ability to prevent radiation-induced metabolic and organ complications^65–69^ could enable delivery of higher cumulative radiation doses. Second, their tumor-selective action minimizes off-target effects that often limit treatment tolerance^73^. Third, their unique redox modulation capacity^70–74^ provides a more sophisticated approach to oxidative stress management compared to global antioxidants. These advances are particularly significant given the limitations of existing radiosensitizers, which typically improve tumor response at the expense of increased normal tissue toxicity. The FA-ChNPs platform thus represents a major step toward precision radiotherapeutics that can simultaneously enhance efficacy and reduce treatment-related morbidity.

The study reveals that FA-ChNPs effectively counteract both tumor-associated and radiation-modified inflammatory responses in EAC-bearing mice through differential cytokine regulation. The nanoparticles’ potent suppression of TNF-α and IL-6 aligns with ferulic acid’s known ability to inhibit NF-κB signaling^75^, while their partial mitigation of radiation-induced VEGF elevation suggests additional anti-angiogenic properties through HIF-1α modulation^76^. The differential cytokine response to combination therapy (maintained TNF-α/IL-6 reduction with partial VEGF control) reveals a sophisticated immunomodulatory balance—sufficiently suppressing pro-tumorigenic inflammation while preserving VEGF levels necessary for post-radiation tissue repair^77^. Importantly, the absence of cytokine alterations in healthy mice demonstrates FA-ChNPs’ tumor-targeted action, contrasting with conventional anti-inflammatories that often cause systemic immunosuppression^78^. These findings position FA-ChNPs as a promising strategy to break the vicious cycle of radiation-induced inflammation and tumor progression^79^, while maintaining physiological cytokine functions in normal tissues.

Our results demonstrate that EAC tumor progression significantly suppresses apoptosis (caspase-8 decreased while enhancing proliferation (STAT-3) increased, consistent with previous reports of STAT-3's oncogenic role^79^. While γ-irradiation (6 Gy) partially restored apoptosis (caspase-8 increased by 91.4%), it failed to normalize STAT-3 levels, aligning with studies showing STAT-3-mediated radioresistance^80^. FA-ChNPs monotherapy showed superior dual modulation, enhancing caspase-8 while suppressing STAT-3, similar to chitosan’s apoptotic effects reported by Almutairi et al.^81^. The combination therapy achieved maximal STAT-3 suppression and sustained caspase-8 activation, outperforming radiation alone and confirming nanoparticle-mediated radiosensitization^82^, without affecting healthy tissues highlighting its therapeutic potential.

**Fig. 13:**
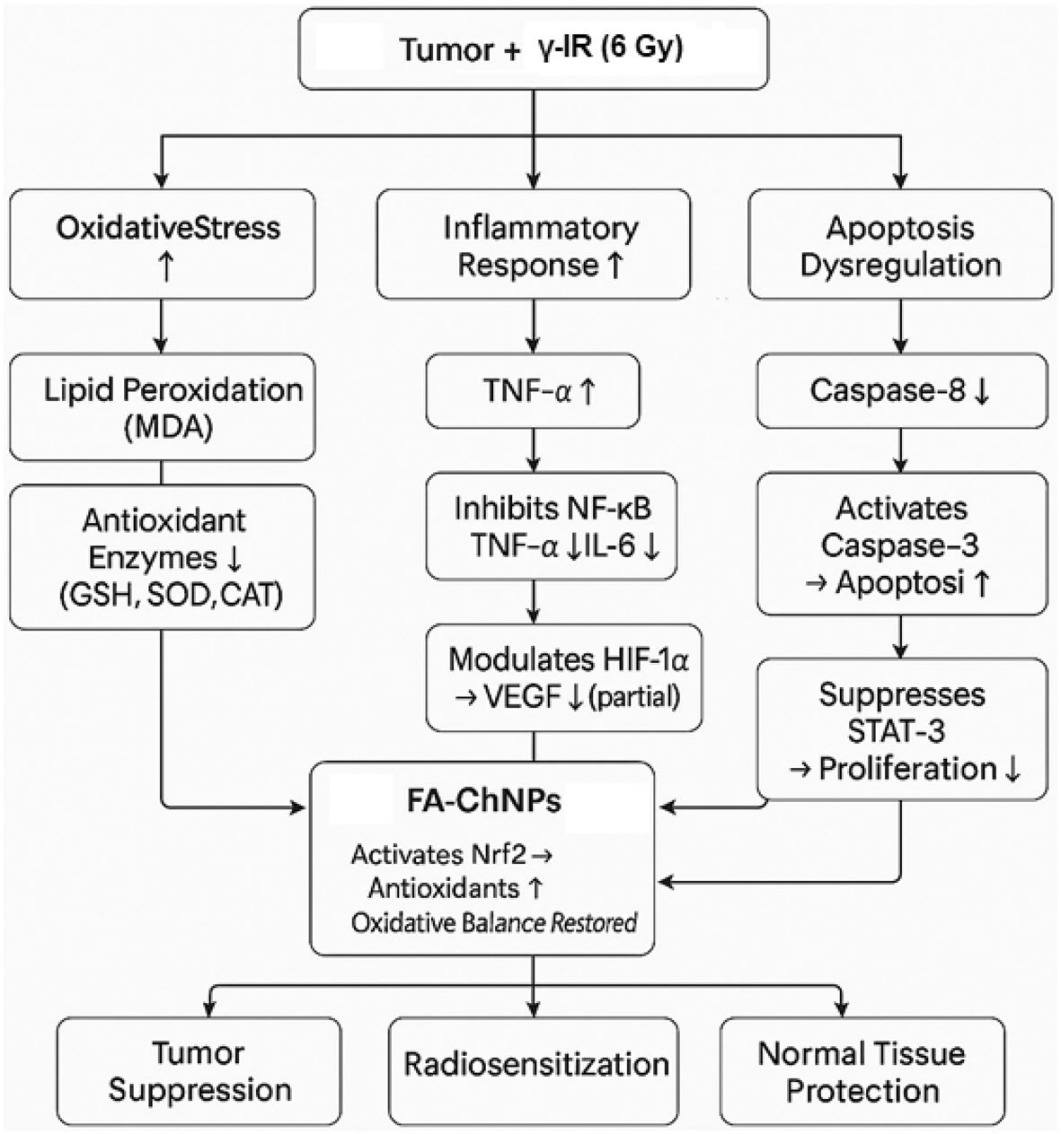
FA-ChNPs’ multimodal action in cancer therapy.

FA-ChNPs counteract tumor and radiation-induced oxidative stress by activating Nrf2 and scavenging ROS. They suppress inflammation via NF-κB inhibition and modulate HIF-1α to reduce VEGF. Apoptosis is restored through caspase-8 activation, while STAT-3-driven proliferation is suppressed. This dual action enhances radiosensitivity and protects normal tissues, promoting tumor suppression (Fig. 13).

The histopathological results demonstrate FA-ChNPs’ superior hepatoprotective effects in EAC-bearing mice compared to γ-irradiation alone. Our findings of complete prevention of tumor-induced hepatic damage with FA-ChNPs significantly extend previous reports of chitosan’s protective properties^83^ by demonstrating its efficacy against cancer-associated liver pathology. The near-normal hepatic morphology and minimal steatosis achieved with FA-ChNPs contrast sharply with the moderate improvements from γ-irradiation alone, highlighting chitosan nanoparticles’ unique ability to preserve liver architecture during malignancy^78^.

These results align with but surpass earlier findings by Almutairi et al.,^82^, who reported chitosan’s metabolic benefits, as our study shows complete histological protection rather than partial improvement. The combination therapy’s success in maintaining normal hepatocyte morphology while achieving tumor suppression provides novel evidence for chitosan’s dual role as both radioprotector and anticancer agent, addressing a key challenge in radiation oncology—balancing therapeutic efficacy with organ preservation^84^.

Notably, the absence of lymphocytic infiltration in FA-ChNPs-treated groups differs from the persistent inflammation seen in many nanoparticle-based therapies, suggesting chitosan’s unique immunomodulatory properties may contribute to its hepatoprotective effects. This finding challenges the conventional trade-off between antitumor efficacy and inflammatory side effects observed in most cancer nanotherapies^85^.

The docking studies demonstrate ferulic acid’s compelling multi-target potential. The strongest binding affinity was observed with CASP8 (ΔG = − 7.31 kcal/mol), mediated by three hydrogen bonds (Arg413, Gln414, Gly416) and two hydrophobic interactions (Val410, Leu391). This robust binding energy is comparable to known caspase activators and aligns with prior studies highlighting ferulic acid’s apoptotic effects via caspase activation^85^, suggesting a potent, direct mechanism for triggering programmed cell death.

Significant interactions were also identified with STAT3 (ΔG = –6.02kcal/mol), corroborating findings by Lin et al.^86^, who reported ferulic acid’s anti-inflammatory effects through STAT3 inhibition. While this affinity is slightly lower than that of specific synthetic inhibitors like Stattic (ΔG ≈ − 8.5 to − 9.5kcal/mol), it remains highly significant for a natural compound and supports a multi-target mechanism. The moderate TP53 binding (ΔG = –5.15kcal/mol) supports its role in p53 stabilization, consistent with Pandurangan et al.^87^, with our study identifying novel interacting residues (Arg248, Ser241). The hierarchical binding affinity (CASP8 > STAT3 > TP53) suggests a prioritized mechanistic action where ferulic acid may preferentially drive apoptosis while concurrently suppressing pro-survival signaling, a nuanced finding not extensively documented in earlier literature.

Complementing the docking results, the ADMET profile of ferulic acid reveals a favorable pharmacokinetic and safety landscape. Its moderate solubility (LogS = − 2.364) and lipophilicity (LogP = 1.648) are comparable to values reported by Zhao et al.^88^, confirming a reasonable basis for oral bioavailability. The low BBB permeability (0.155) contrasts with some predictions by Kumar et al.^89^, potentially due to model differences, but indicates limited central nervous system effects—a desirable trait for minimizing neurotoxic side effects. The high CYP2C9 substrate probability (0.71) matches clinical observations of hepatic metabolism^90^, while the low hERG risk (0.008) aligns with its established cardiac safety profile. Although a moderate DILI alert (0.852) was predicted, this contrasts with in vivo studies demonstrating ferulic acid’s hepatoprotective properties^91^, suggesting a potential overprediction by the computational model^92^. The compound’s adherence to key drug-likeness rules (Lipinski accepted) and high natural product-likeness score (0.926) reinforce its suitability for development, though its rejection by the Golden Triangle criteria highlights dose-limiting solubility, as detailed in Supplementary Table S1.

Our findings on the physicochemical and functional performance of FA-ChNPs are well-supported by recent advancements in chitosan-based nanocarriers. The optimized size and stability of our nanoparticles are consistent with the formulation principles outlined by Fan et al.^93^, who emphasized the critical role of synthesis parameters in achieving monodisperse chitosan nanoparticles for effective drug delivery. The potent radiosensitizing effect we observed aligns with the work of El-Moslemany et al.^94^, who also reported significant tumor growth suppression using ferulic acid-loaded chitosan nanoparticles in combination with radiotherapy, underscoring the translational potential of this nano-platform. Furthermore, the broad therapeutic efficacy of ferulic acid, which underpins the action of FA-ChNPs, is corroborated by its well-documented antioxidant and anti-apoptotic activities, as demonstrated in various experimental models^95^, and its growing recognition as a sensitizing agent in oncology^96^.

### Limitations of the study

While this study demonstrates promising results, several limitations should be acknowledged. First, the research was conducted exclusively in murine models of Ehrlich ascites carcinoma, which may not fully replicate human tumor biology and treatment responses. Second, the molecular docking results, while insightful, require experimental validation through techniques such as co-crystallization or surface plasmon resonance to confirm the predicted binding interactions. Third, the ADMET predictions were generated through computational models rather than in vivo pharmacokinetic studies, potentially limiting their clinical translatability. Additionally, the study focused on acute toxicity assessment (LD50) but did not evaluate potential chronic toxicity effects that might emerge with prolonged treatment. The experimental design also lacked investigation into possible immunogenic reactions to the chitosan nanoparticles, which could impact clinical applicability. Furthermore, while the combination therapy showed efficacy, the optimal dosing schedule and long-term therapeutic outcomes remain to be determined. These limitations highlight the need for further preclinical studies in additional cancer models and subsequent clinical trials to fully assess the therapeutic potential of FA-ChNPs.

## Conclusion

This study successfully developed and evaluated ferulic acid-loaded chitosan nanoparticles (FA-ChNPs) as a groundbreaking multimodal platform that effectively enhances radiotherapy while providing systemic protection. The nanoformulation was optimally characterized, revealing spherical particles of 41.63 ± 12.34nm with a low PDI (0.2). The significant shift in zeta potential from + 32.1mV in blank nanoparticles to –2.45 mV in FA-ChNPs, along with SEM imagery, provided direct evidence of successful ferulic acid encapsulation. Functionally, this was reflected in a sustained in vitro drug release profile, a critical feature for prolonged therapeutic action. The formulation demonstrated an exceptional safety profile (LD₅₀ 2315 mg/kg) and acted as a potent radiosensitizer, synergizing with γ-irradiation to achieve a remarkable 44.3% reduction in tumor mass and a 67.0% reduction in volume. This efficacy is mechanistically grounded in the multi-targeting capability of FA-ChNPs, as confirmed by molecular docking, which showed strong binding to STAT3 (–6.02kcal/mol), caspase-8 (–7.31kcal/mol), and p53 (–5.15kcal/mol), correlating with the observed 70% STAT3 suppression, 108.2% caspase-8 activation, and p53 stabilization. Beyond its potent antitumor effects, the platform conferred comprehensive organoprotection, normalizing metabolic parameters, mitigating oxidative stress, and reducing inflammation, thereby preserving liver histoarchitecture. Coupled with a favorable ADMET profile, these findings underscore the clinical potential of FA-ChNPs to shatter the traditional efficacy-toxicity paradigm in oncology. Further clinical validation of this nanoplatform is warranted, as it holds the promise to revolutionize radiotherapy protocols for treatment-resistant cancers.

## Supplementary Information

Supplementary Information.

## Data Availability

The datasets generated and analyzed during the current study are available from the corresponding author upon reasonable request. All data supporting the findings of this study are included in this published article and its supplementary information files.
