# Supplementary material for: Ferulic acid-loaded chitosan nanoparticles enhance radiotherapy efficacy via STAT3 suppression and caspase-8/p53 activation in Ehrlich ascites carcinoma
Source: Sci Rep. 2025 Dec 1;15:42910. doi: 10.1038/s41598-025-27352-8 (PMC12672582; doi:10.1038/s41598-025-27352-8)
Supplement: Supplementary file 1 — Supplementary Information. [file 41598_2025_27352_MOESM1_ESM.docx]

**ADMET analysis of Ferulic acid**

ADMETlab2.0 (6) server was utilized to predict the ADMET properties of Ferulic acid.

The ADMET parameters of Ferulic acid was input into the ADMETlab3.0 server and various pharmacokinetic parameters like aqueous solubility, lipophilicity, intestinal absorption, BBB permeability, plasma protein binding, cytochrome P450 metabolism, drug-drug interaction potential, toxicity endpoints and drug likeness scores were calculated using pre-trained predictive models.

**Supplementary Table S1.**

| **ADMET parameters** | **Ferulic acid** |
| --- | --- |
| LogS | -2.364 |
| LogD | 1.807 |
| LogP | 1.648 |
| Pgp-inh | 0.0 |
| Pgp-sub | 0386 |
| HIA | 0.014 |
| F(20%) | 0.004 |
| F(30%) | 0.971 |
| Caco-2 | -4.989 |
| MDCK | 0.0 |
| BBB | 0.155 |
| PPB | 78.09 |
| VDss | 0.29 |
| Fu | 10.15 |
| CYP1A2-inh | 0.091 |
| CYP1A2-sub | 0.234 |
| CYP2C19-inh | 0.035 |
| CYP2C19-sub | 0.051 |
| CYP2C9-inh | 0.063 |
| CYP2C9-sub | 0.71 |
| CYP2D6-inh | 0.044 |
| CYP2D6-sub | 0.24 |
| CYP3A4-inh | 0.039 |
| CYP3A4-sub | 0.04 |
| CL | 6.856 |
| T12 | 0.916 |
| hERG  Blockers | 0.008 |
| H-HT | 0.399 |
| DILI | 0.852 |
| AMES Toxicity | 0.086 |
| ROA | 0.065 |
| FDAMDD | 0.019 |
| SkinSen | 0.779 |
| Carcinogenicity | 0.702 |
| EC | 0.854 |
| EI | 0.989 |
| Respiratory | 0.397 |
| BCF | 0.482 |
| IGC50 | 3.265 |
| LC50 | 3.686 |
| LC50DM | 4.229 |
| NR-AR | 0.577 |
| NR-AR-LBD | 0.659 |
| NR-AhR | 0.197 |
| NR-Aromatase | 0.039 |
| NR-ER | 0.387 |
| NR-ER-LBD | 0.078 |
| NR-PPAR-gamma | 0.439 |
| Skin_Sensitization | 0.609 |
| Acute_Aquatic_Toxicity | 0 alerts |
| Toxicophores | 0.0 |
| QED | 0.715 |
| Synth | 1.869 |
| Fsp3 | 0.1 |
| MCE-18 | 7.0 |
| Natural Product-likeness | 0.926 |
| Alarm NMR | 2 alerts |
| BMS | 0 alerts |
| Chelating | 1 alerts |
| PAINS | 0 alerts |
| Lipinski | Accepted |
| Pfizer | Accepted |
| GSK | Accepted |
| Golden Triangle | Rejected |
